# Integrated transcriptomic and metabolomic profiling reveals the flower color formation mechanism of alfalfa different purple flowers

**DOI:** 10.3389/fpls.2026.1786493

**Published:** 2026-03-23

**Authors:** Hongqi Du, Changsong Feng, Zhiyan Liang, Zhiguo Lou, Lei Liu

**Affiliations:** Institute of Animal Husbandry, Henan Academy of Agricultural Sciences, Zhengzhou, Henan, China

**Keywords:** alfalfa, anthocyanin, flower, pigment glycoside, purple color

## Abstract

**Background:**

Flower color plays a crucial role in plant identification, evaluation, and evolution, and it is directly or indirectly associated with the agronomic characteristics of plants. Alfalfa (*Medicago sativa L.*) is an important leguminous forage grass, it exhibits a continuous gradient of purple flowers, but the regulatory mechanisms underlying this quantitative variation remain poorly understood.

**Methods:**

We integrated metabolomics and transcriptomics analyses to investigate five alfalfa flower types: white (BH) and four shades of purple (ZH1-ZH4, from light to dark). Anthocyanin and carotenoid contents were quantified by LC-ESI-MS/MS. Differentially accumulated pigments were subjected to KEGG enrichment and statistical analysis. RNA-seq reads were mapped to the Medicago reference genome, and differentially expressed genes (DEGs) were identified using DESeq2. Common significant DEGs (CSDEGs) among the four purple-versus-white comparisons were clustered to identify expression trends. Quantitative Real-time PCR (qRT-PCR) was performed to validate the transcriptome data, and an O2PLS model was used to integrate metabolomic and transcriptomic datasets.

**Results:**

Metabolomics analysis revealed that delphinidin-3, 5-O-diglucoside and malvidin-3, 5-O-diglucoside were the two most abundant anthocyanins, and their contents increased steadily from ZH1 to ZH4. The ratio of blue to red pigments decreased along the same gradient. Transcriptomics identified 580 CSDEGs, of which 108 showed a regular trend across the five flower types, and 24 were transcription factors, with MYB and bHLH being the most represented. CSDEGs are involved in multiple processes such as stimulation of the internal and external cellular environment, pigment synthesis and regulation, pigment transport and deposition. Notably, 29 genes in CSDEGs were definitively involved in the anthocyanin biosynthesis chain, and some important candidate genes regulating the formation of purple color in alfalfa flowers were identified. Joint analysis of the two datasets revealed that the relative expression levels of two novel genes (MSTRG.59017, MSTRG.14861) were positively correlated with the contents changes of delphinidin-3, 5-O-diglucoside or malvidin-3, 5-O-diglucoside in BH, ZH1, ZH2, ZH3, and ZH4.

**Conclusion:**

Anthocyanins are the dominant pigments responsible for the purple coloration of alfalfa flowers. Delphinidin and malvidin derivatives, especially delphinidin-3, 5-O-diglucoside and malvidin-3, 5-O-diglucoside, are the key molecules driving the purple gradient, as well as the proportional relationship between blue and red pigments. Genes responding to the intracellular and extracellular environments influencing the purple formation of alfalfa flowers, involved in transcriptional, translational and post-translational regulation of anthocyanin biosynthesis, and involved in synthesis, transport and deposition of anthocyanin were screened out. We confirmed that there should be key genes specific to the synthesis of each anthocyanin glycoside, which will be the focus of future research. This study has established a theoretical foundation for explaining the different purple color of alfalfa flowers. This study enriches the theory of plant flower color formation and provides a theoretical basis for alfalfa flower color improvement and molecular breeding.

## 1 Introduction

Flower color is a vital morphological trait of plants that plays a crucial role in plant identification, evaluation, and evolution (Davies et al., 2012; Sobel and Streisfeld, 2013). Variation in flower color serves a significant ecological function by attracting pollinators, thereby influencing the reproductive success and seed yield of flowering plants (Meng et al., 2019; Zhang, 1989). Additionally, flower color can protect plants and their reproductive organs from UV damage, pests, and pathogens, and it helps maintain an appropriate energy balance in flower tissues under diverse light conditions (Steyn et al., 2002; Winkel-Shirley, 2001). Flower color is directly or indirectly associated with the agronomic characteristics of plants. Consequently, it has been used as an indicator character to study its relationship with other traits and is widely applied in the routine breeding of plant varieties (Hanumappa et al., 2007). For example, flower and leaf colors can serve as diagnostic aids in alfalfa varietal identification (Liu et al., 2023).

Regarding the formation mechanism of plant flower color, researchers have made several key findings: (1) The material basis of plant color formation is various pigments, such as flavonoids, carotenoids, chlorophyll, alkaloids, and so on. (2) The synthetic pathways of each pigment and the important genes involved in their synthesis have been identified. (3) Gene expression and pigment synthesis are primarily regulated by transcription, post-transcription, epigenetics, hormones, and other factors. (4) The color of pigments is influenced by internal environmental factors (e.g., pH, metal ions and sugars) and external environmental factors (e.g., light, temperature, water, soil nutrients, and organisms). Different pigments exhibit distinct colors, and the same pigment can exhibit different colors under varying environmental conditions (Zhao et al., 2005; Tanaka et al., 2008; Yoshida, 2024; Zhao and Tao, 2015). (5) The spatio-temporal combination of different pigment types and their contents ultimately determines flower color (Yasuda, 1989; Harborne, 1993). Pigment color has a quantitative effect. However, it is not exactly the same as the color of the pigments contained in the petals. Anthocyanins are responsible for the various hues of pink, red, purple, blue, and red-purple colors in flowers. In anthocyanins, delphinins, petunins, and malvins display blue-purple colors, pelargonins show a brick-red color, and cyanins and peonins present purplish-red colors (Yasuda, 1989; Hugo and Tinothy, 1991). Carotenoids (carotene, xanthophyll) exhibit colors such as yellow, orange, red, and purple (Goodwin and Britton, 1988).

Alfalfa is an important leguminous forage grass, with widely cultivated species including *Medicago sativa*, *Medicago falcata*, *Medicago lupulina*, and *Medicago varia*. Alfalfa flower colors are diverse, encompassing light purple, purple, dark purple, reddish-purple, bluish-purple, light yellow, yellow, dark yellow, white, milky white, yellow-white, and various variegated flowers (Lesins, 1956; Wang et al., 2006; Wu, 2008; Yun et al., 2004; Yang, 2012). The diverse flower colors in alfalfa are partly due to the fact that *M. sativa ssp. sativa* and *M. sativa ssp. falcata* can freely cross without reproductive isolation from each other. Alfalfa plants with different flower colors have significantly distinct agronomic traits, such as plant height, leaf area, nutritional quality, and stress resistance (By Roberts and Freeman, 1907; Julier et al., 2000). For instance, *Medicago falcata* is known for its strong cold and drought resistance (Sun et al., 2022; Li et al., 2023), whereas *Medicago sativa* has significant advantages in agronomic traits (Zhang et al., 2022; Yang et al., 2020; Wang et al., 2003). Therefore, understanding the mechanisms of different flower color formation in alfalfa and identifying related key genes would be highly informative for the utilization and nutritive research on alfalfa germplasms, molecular genetic improvement of related traits of alfalfa, and the creation of novel alfalfa germplasms. However, currently, there is relatively limited research on the mechanisms of different flower color formation in alfalfa.

In recent years, some progress has been made in understanding the formation mechanism of alfalfa flower color. Variety, soil pH, soil fertility, light exposure, and seed source have all been found to influence the purple color of alfalfa flowers (Sheridan and Mckee, 1970). Research on the inheritance of alfalfa flower color has revealed that the purple color is due to a sap-soluble anthocyanin pigment, specifically malvin. The production of malvin is conditioned by two complementary genes. In the absence of either of these genes, *M. sativa* flowers are white. Additionally, flowers of different shades of purple, violet, or blue may contain other members of the anthocyanin series (Twamley, 1955). The intensity of purple pigmentation appears to be controlled to some extent by intensification factors, but it is also probable that the number of dominant alleles present at the complementary loci is a determining factor. Three factor pairs are believed to control purple flower color, which can be designated as P1p1P2p2P3p3 (Weihing, 1948). Three anthocyanins have been found in all flowers with blue or purple color and are inherited as a genetic unit under the control of a single dominant gene P. A fairly high level of yellow anthoxanthin pigment is present in all flowers examined, including white flowers, suggesting that plants in this population are homozygous for a basic color factor (C) (Cooper and Elliott, 1965).

Recent studies have made important progress in dissecting the basis of floral pigmentation variation in alfalfa. Anthocyanins and carotenoids are the main coloring substances in the flower color formation of purple alfalfa and yellow alfalfa, respectively. Alfalfa flowers contain three anthocyanin pigments: the 3, 5 diglucosides of delphinidin, petunidin, and malvidin. The anthocyanin derivatives delphinidin-3, 5-O-diglucoside or malvidin and petunidin glycoside derivatives are the key pigments regulating the flower color formation of purple alfalfa. The carotenoid derivative lutein is the key pigment regulating the flower color formation of yellow alfalfa. A large number of potential regulatory factors for anthocyanin in alfalfa have been obtained, and key genes related to alfalfa flower color formation have been chosen. These studies laid a solid foundation for understanding the interspecific color differences of alfalfa flowers (Cooper and Elliott, 1964; Duan et al., 2020; Pan et al., 2021; Huang et al., 2024; Wen et al., 2025).

Floral anthocyanin biosynthesis largely depends on the conserved MYB-bHLH-WD40 (MBW) activation complex and MYB repressors that interact hierarchically with the MBW complex (Li et al., 2020), transcription factors (TFs) of the MYB and bHLH families play a vital role in the regulation of anthocyanin biosynthesis (Schwinn et al., 2006; Zhou et al., 2024; Wang et al., 2019). Notably, these TFs also play a positive role in regulating anthocyanin biosynthesis in alfalfa. CsMYB5–1 and CsMYB5–2 differently regulate anthocyanins/proanthocyanidins in alfalfa flowers (Zheng et al., 2019). The MYB transcription factor LAP1 induces massive accumulation of anthocyanin pigments, including multiple glycosidic conjugates of cyanidin (Peel et al., 2009). The MYB transcription factor WP1 interacts with MtTT8 and MtWD40–1 to form an MBW complex, which enhances the transcriptional activation ability of WP1 on anthocyanin and carotenoid biosynthesis genes (Meng et al., 2019), and the bHLH transcription factor MtTT8 regulates a subset of genes involved in PA and anthocyanin biosynthesis in Medicago truncatula (Li et al., 2016).

However, existing studies mainly focus on the qualitative differences between distinct color categories, while the regulatory mechanism underlying the continuous variation of purple intensity (i.e., from light purple to deep purple) within the purple flower color system remains poorly understood. The gradient variation of purple color is a more refined phenotypic trait than the intercategory color difference, which is likely regulated by the quantitative changes of key genes and metabolites rather than the presence or absence of specific components.

This research project is designed to take white flowers and purple flowers with a gradient from light to dark of alfalfa as the research subjects. By employing metabolomic and transcriptomic approaches, this study aims to explore, from the perspective of the continuous gradation of purple, the pigments that exhibit regular changing trends and the dominant pigments in the measured pigment content, and how different anthocyanins are superimposed to form the purple color. It will also conduct research and analysis on the common genes with significant differences between purple and white flowers, genes with regular expression trends, and the Gene Ontology (GO) and KEGG Orthology (KO) terms enriched by these genes.Ultimately, through a comprehensive analysis of these findings, the mechanism underlying the formation of purple alfalfa flowers will be elucidated.

## 2 Materials and methods

### 2.1 Materials and sampling

White flowers and four types of purple flowers, ranging from light purple to deep purple, were collected from the alfalfa germplasm resource garden at the Henan Modern Agricultural Science and Technology Experimental Demonstration Base of Henan Academy of Agricultural Science, Xinxiang, China (35°0′19.16″N, 113°42′16.79″E).

Three biological replicates were taken for each flower type. Each replicate consisted of five inflorescences of the same color, which were thoroughly mixed. The three replicate samples of white flowers were designated as BH1, BH2, and BH3. The replicates of the four types of purple flowers were sequentially named ZH1-1, ZH1-2, ZH1-3; ZH2-1, ZH2-2, ZH2-3; ZH3-1, ZH3-2, ZH3-3; and ZH4-1, ZH4-2, ZH4-3(collectively referred to as ZHs).

Each sample was divided into two parts: one half was used for transcriptome analysis, and the other half was allocated for metabolome analysis.

### 2.2 Metabolome analysis

#### 2.2.1 Sample preparation and extraction

Each sample was freeze-dried, ground into powder (30 Hz, 1.5 min), and stored at -80°C until use. A total of 50 mg of powder was weighed and extracted with 0.5 mL of methanol/water/hydrochloric acid (500:500:1, V/V/V). The extract was vortexed for 5 min, sonicated for 5 min, and centrifuged at 12, 000 g at 4°C for 3 min. The residue was re-extracted by repeating the above steps under the same conditions. The supernatants from the two extractions were combined, collected, and filtered through a 0.22 µm membrane filter (Anpel) prior to LC-MS/MS analysis.

#### 2.2.2 UPLC-ESI-MS/MS analysis

The sample extracts were analyzed using a UPLC-ESI-MS/MS system, consisting of an ExionLC™ AD UPLC (https://sciex.com.cn/) and an Applied Biosystems 6500 Triple Quadrupole MS (https://sciex.com.cn/).

Specifically, linear ion trap (LIT) and triple quadrupole (QQQ) scans were acquired on QTRAP^®^ 6500+ LC-MS/MS System(a triple quadrupole-linear ion trap mass spectrometer, QTRAP), which was equipped with an ESI Turbo Ion-Spray interface, the system operated in positive ion mode and was controlled by Analyst 1.6.3 software (Sciex).

#### 2.2.3 Detection of anthocyanins and carotenoids

Anthocyanins and Carotenoids were analyzed using scheduled multiple reaction monitoring (MRM). Data acquisitions were performed using Analyst 1.6.3 software (Sciex). Multiquant 3.0.3 software (Sciex) was used to quantify all metabolites. Mass spectrometer parameters, including the declustering potentials (DP) and collision energies (CE) for individual MRM transitions, were optimized. A specific set of MRM transitions was monitored for each period in accordance with the metabolites eluted within that period.

Anthocyanins and Carotenoids contents were detected by MetWare (http://www.metware.cn/) based on the AB Sciex QTRAP 6500 LC-MS/MS platform.

#### 2.2.4 Hierarchical cluster analysis

The hierarchical cluster analysis (HCA) results of samples and metabolites were presented as heatmaps with dendrograms. HCA was carried out using the R package pheatmap. For HCA, normalized signal intensities of metabolites (after unit variance scaling) were visualized as a color spectrum.

#### 2.2.5 Differential anthocyanins and carotenoids selected and their KEGG enrichment analysis

Significantly regulated anthocyanins and carotenoids between groups were determined based on absolute Log_2_ Fold Change (Log_2_FC). Identified anthocyanins and carotenoids were annotated using the KEGG compound database (http://www.kegg.jp/kegg/compound/), and then mapped to the KEGG Pathway database (http://www.kegg.jp/kegg/pathway.html). Pathways with significantly regulated anthocyanins and carotenoids were fed into metabolite sets enrichment analysis (MSEA), and their significance was determined by hypergeometric test p-values.

#### 2.2.6 Statistical analysis of pigment content

The anthocyanins and carotenoids in BH, ZH1, ZH2, ZH3, and ZH4 samples were statistically analyzed and categorized by their content levels: < 1 µg/g, > 1 µg/g and < 10 µg/g, > 10 µg/g and < 100 µg/g, and > 100 µg/g.

The colors of anthocyanin glycosides and carotenoids were determined through literature retrieval, and the pigment content corresponding to each color was counted using Excel. The ratio of blue and red pigment content was calculated. The proportion of pigments with the highest proportion in blue and red pigments was identified, and the ratio of the two was counted.

### 2.3 Transcriptome analysis

#### 2.3.1 RNA extraction, library construction, sequencing and differential expression genes analysis

Total RNA was extracted from each sample using the Trizol reagent kit (Invitrogen, Carlsbad, CA, USA) according to the manufacturer’s protocol. The RNA quality was assessed using an Agilent 2100 Bioanalyzer (Agilent Technologies, Palo Alto, CA, USA) and verified via RNase-free agarose gel electrophoresis. After total RNA extraction, mRNA was enriched using Oligo(dT) beads. The enriched mRNA was then fragmented into short fragments using a fragmentation buffer and reverse-transcribed into cDNA using the NEB Next Ultra RNA Library Prep Kit for Illumina (NEB#7530, New England Biolabs, Ipswich, MA, USA). The purified double-stranded cDNA fragments were end-repaired, had an A base added, and were ligated to Illumina sequencing adapters. The ligation reaction was purified using AMPure XP Beads (1.0X) and amplified via polymerase chain reaction (PCR). The resulting cDNA library was sequenced on the Illumina NovaSeq 6000 platform by Gene Denovo Biotechnology Co. (Guangzhou, China), generating 150 bp paired-end reads.

To obtain high-quality clean reads, further filtering was conducted using fastp (version 0.18.0). An index of the reference genome was constructed, and paired-end clean reads were mapped to the reference genome (https://figshare.com/projects/whole_genome_sequencing_and_assembly_of_Medicago_sativa/66380) using HISAT2.2.4 with default parameters. Reads that did not map to the reference genome were blasted against the NCBI database, and these unaligned genes were designated as “MSTRG.****”.

Mapped genes, blasted genes, annotated genes, unannotated genes, and transcription factors were statistically distinguished. Additionally, three types of transcription factors-MYB, bHLH, and WD40-, were specifically searched within the transcription factor set.

The mapped or blasted reads of each sample were assembled using StringTie v1.3.1 in a reference-based approach. To standardize gene expression levels, the transcript value of each Unigene was standardized to the fragments per kilobase of transcript per million fragments mapped (FPKM), which was used to represent the level of gene expression.

Correlation analysis was performed using R. Principal component analysis (PCA) was conducted using the R package gmodels (http://www.r-project.org/).

RNA differential expression analysis was performed using DESeq2 software to compare BH with each of ZH1, ZH2, ZH3, and ZH4. Genes with a false discovery rate (FDR) below 0.05 and an absolute fold change ≥ 2 were considered significant differentially expressed genes (SDEGs). SDEGs from all four comparisons (BH vs. ZH1, BH vs. ZH2, BH vs. ZH3, BH vs. ZH4) were grouped together as total significant differentially expressed genes (TSDEGs). The common genes among these four sets of SDEGs were identified as common significant differentially expressed genes (CSDEGs). Furthermore, the unannotated genes in CSDEGs and those annotated in the NR database were counted, and the relative expression levels of the unannotated genes in NR database were presented in tabular form.

CSDEGs were first mapped to GO terms in the Gene Ontology database (http://www.geneontology.org/), and the number of genes associated with each term was calculated. Significantly enriched GO terms in CSDEGs compared to the genome background were identified using the hypergeometric test. The calculated p-values were corrected using the FDR method, with FDR ≤ 0.05 as the threshold. GO terms meeting this condition were defined as significantly enriched in CSDEGs. Subsequently, the enrichment of GO terms in the Molecular Function, Cellular Component, and Biological Process categories of CSDEGs was statistically analyzed.

In KEGG database, pathway enrichment analysis was conducted to identify significantly enriched metabolic pathways or signal transduction pathways in CSDEGs compared to the whole genome background. The calculated p-values were corrected using the FDR method, with FDR ≤ 0.05 as the threshold. Pathways meeting this condition were defined as significantly enriched in CSDEGs.

Based on KEGG data, the anthocyanin biosynthesis chain was constructed by backward derivation from the anthocyanin biosynthesis pathway. These pathways were checked in the pathway enrichment analysis results of CSDEGs, and the CSDEGs involved in these pathways, along with their expression levels in ZH1, ZH2, ZH3, and ZH4 relative to BH, were presented in tabular form.

The functions of CSDEGs not enriched in KEGG pathways were understood by searching the UniProt and pubmed database, and some important genes that may be involved in flower color formation were selected.

#### 2.3.2 Expression trend analysis of CSDEGs

Trend analysis (Series Test of Cluster) is an essential method in gene expression analysis. Based on the FPKM values of CSDEGs, cluster analysis was performed using Mfuzz R package, and CSDEGs were classified according to their expression trends. The unannotated CSDEGs in the reference genome were counted and analyzed in profiles 9, 8, and 0. Additionally, the expression trends of CSDEGs involved in the anthocyanin biosynthesis chain pathways were shown in tabular form.

#### 2.3.3 Validation of differentially expressed genes

Twelve genes from CSDEGs involved in anthocyanin glycoside synthesis chain were selected for validation via qRT-PCR. The same batch of samples used in transcriptome sequencing was employed for qPCR detection.The first strand cDNA for each sample was synthesized from 1 μg total RNA using Revert Aid First Strand cDNA Synthesis Kit (Fermentas) following the manufacturer’s instruction and diluted 10-fold before performing PCR. The qRT-PCR primers and corresponding genes are shown in **Supplementary Table 1**. *Medicago sativa* putative glyceraldehyde-3-phosphate dehydrogenase (MsGAPDH) was selected as the reference gene for normalization. The mRNA abundance of each gene in all samples was detected using the Roche SYBR Green fluorescent dye method and a ROCHE LightCycler^®^ 96 Real-Time Fluorescent Quantitative PCR Instrument (Roche Diagnostics GmbH, Manheim, Germany), and SYBR Green PCR Master Mix (TaKaRa, Shiga, Japan). The amplification program was as follows: 90 s at 95°C, followed by 40 cycles of 95°C for 5 s and 60°C for 30 s, the dissolution curve of the amplified product was analyzed from 65°C to 95°C. The Relative expression levels were calculated relative to a calibrator samples using the formula 2^−ΔΔCt^, and the values were presented as mean ± standard deviation (Livak and Schmittgen, 2001). Significant differences were assessed by one-way ANOVA in SPSS 22 (IBM Corp., USA). The statistical analysis results of the relative expression abundance of these genes mRNA obtained by qRT-PCR were compared with their transcriptome data. Finally, their qRT-PCR data and transcriptome data were used to construct a bar chart in GraphPad prism 5 (GraphPad Software, Inc., USA).

### 2.4 Combined transcriptomic and metabolomic analysis

Anthocyanins synthesized via the ko00942, ko00941, and ko00906 pathways were selected by comparing the KEGG enrichment analysis results of transcriptomic and metabolomic datasets. Significant differentially genes involved in the synthesis of these pigments were identified. The functions of these genes were understood by searching the **Supplementary Materials** and the KEGG database. Moreover, their relative expression trends in BH, ZH1, ZH2, ZH3, and ZH4 were analyzed to determine whether they promote or inhibit pigment synthesis.

The Bidirectional orthogonal projections to latent structures (O2PLS) model was constructed based on gene expression and metabolite abundance data. The associated gene and metabolite sets were predicted by constructing the O2PLS model. For clarity, only unannotated genes that are significantly associated with metabolites are shown here.

## 3 Results

### 3.1 Different color flower

**Figure 1:**
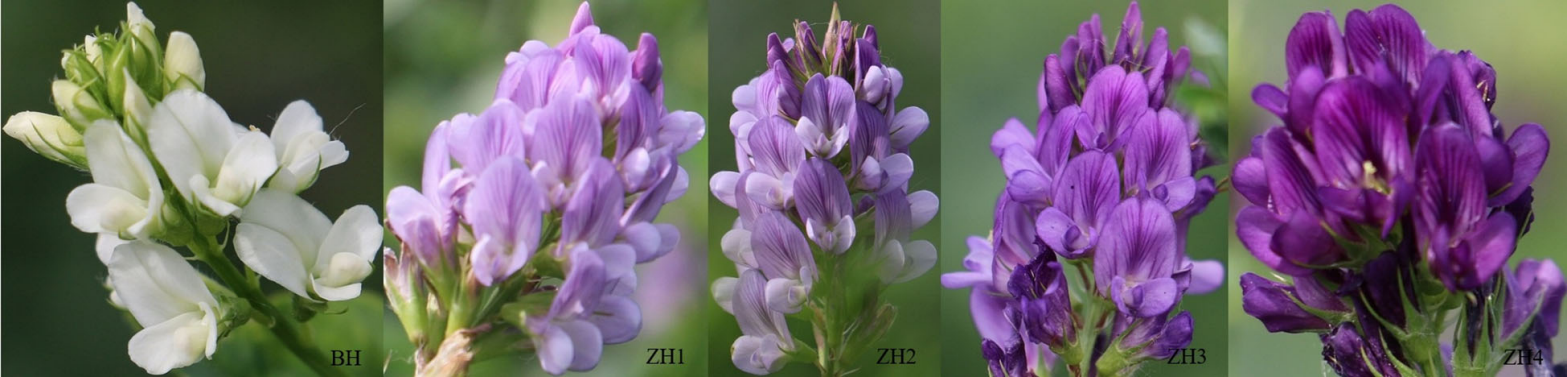
White flowers and flowers in different shades of purple.

**Figure 1** shows five types of alfalfa flowers in different colors. Specifically, from left to right, the colors of the flowers gradually transition from white to light purple and then to dark purple.

### 3.2 Metabolome results

#### 3.2.1 Detection results of anthocyanins and carotenoids in samples

The contents of 64 anthocyanins in BH, ZH1, ZH2, ZH3, and ZH4 are shown in **Supplementary File S1**. Of these 64 anthocyanins, 37 were detected in white flowers, whereas 60 were detected in purple flowers. Notably, Among the 64 anthocyanins detected, eleven anthocyanins in alfalfa flowers had contents greater than 1 µg/g, and the contents of delphinidin-3, 5-O-diglucoside and malvidin-3, 5-O-diglucoside ranked first and second. The content of delphinidin-3, 5-O-diglucoside in purple flowers exceeded 1, 000 µg/g (its content in white flowers was only 7.99µg/g), and the content of malvidin-3, 5-O-diglucoside in purple flowers exceeded 260 µg/g (its content in white flowers was only 6.03 µg/g). Percentages of delphinidin-3, 5-O-diglucoside in the total detected anthocyanins are in order as 13.31%, 76.09%, 71.26%, 72.01%, 67.28% in BH, ZH1, ZH2, ZH, ZH4. Correspondingly, percentages of Malvidin-3, 5-O-diglucoside in the total detected anthocyanins are in order as 13.84%, 16.29%, 19.91%, 23.05%, 26.58%. The combined percentage of Delphinidin-3, 5-O-diglucoside and Malvidin-3, 5-O-diglucoside in the total detected anthocyanins are in order as 27.15%, 92.38%, 91.17%, 95.06%, 93.86%. Furthermore, the proportion of delphinidin-3, 5-O-diglucoside among all detected delphinidin glycosides detected and malvidin-3, 5-O-diglucoside among all detected malvidin glycosides in the samples was above 96.5% and 71.5%, respectively (**Table 1**).

**Table 1:** abundance of Delphinidin-3, 5-O-diglucoside, Malvidin-3, 5-O-diglucoside in samples and their proportion.

Pigments	Pigment abundance and proportion in samples				
	BH	ZH1	ZH2	ZH3	ZH4
Delphinidin-3, 5-O-diglucosides (ug/g)	8.99	1236.20	1689.85	2703.79	2961.74
Delphinidin glycosides (ug/g)	9.27	1261.11	1716.66	2747.78	3027.64
Malvidin-3, 5-O-diglucosides (ug/g)	9.35	264.67	472.23	865.67	1170.26
Malvidin glycosides (ug/g)	13.00	328.16	599.45	963.97	1326.35
Anthocyanidins (ug/g)	67.56	1205.18	2371.37	3755.00	4402.19
Percentage of Delphinidin-3, 5-O-diglucoside in Anthocyanidins (%)	13.31	76.09	71.26	72.01	67.28
Percentage of Malvidin-3, 5-O-diglucoside in Anthocyanidins (%)	13.84	16.29	19.91	23.05	26.58
Percentage of Delphinidin-3, 5-O-diglucoside and Malvidin-3, 5-O-diglucoside in Anthocyanidins (%)	27.15	92.38	91.17	95.06	93.86
Percentage of Delphinidin-3, 5-O-diglucoside in Delphinidin glycosides (%)	96.97	98.02	98.44	98.40	97.82
Percentage of Malvidin-3, 5-O-diglucoside in Malvidin glycoside (%)	71.89	80.65	78.78	89.80	88.23

The contents of 68 carotenoids in BH, ZH1, ZH2, ZH3, and ZH4 are shown in **Supplementary File S2**. Of these 68 carotenoids, 38 were detected in white flowers, while 46 were detected in purple flowers. Fourteen carotenoids in alfalfa flowers had contents greater than 1 µg/g, of which 7 carotenoids’s content in BH is more than that in ZHs. Five carotenoids in alfalfa flowers had contents greater than 5 µg/g, and their contents in BH are more than that in ZHs. Additionally, lutein was the most abundant carotenoid in each sample, with contents ranging from 52 to 114 µg/g.

#### 3.2.2 Statistical analysis of pigment content in different colors

According to the statistics of color categories, the content of pigments corresponding to each color is detailed in **Table 2**. Among all colors pigments, blue pigment had the highest content, with levels in BH, ZH1, ZH2, ZH3, and ZH4 being 8.27 µg/g, 1232.07 µg/g, 1693.08 µg/g, 2723.37 µg/g, and 3014.84 µg/g, respectively. The content of red pigment in BH, ZH1, ZH2, ZH3, and ZH4 was 9.77 µg/g, 327.15 µg/g, 583.75 µg/g, 930.48 µg/g, and 1297.52 µg/g, respectively. Notably, the ratio of blue pigment content to red pigment content showed a gradually decreasing trend in ZH1, ZH2, ZH3, and ZH4.

**Table 2:** Statistical analysis of pigment content in different colors.

Pigments	Pigments color	Pigment abundance in samples (ug/g)				
		BH	ZH1	ZH2	ZH3	ZH4
Procyanidin	colorless	0.10	0.26	0.07	0.10	0.05
Flavone	colorless	45.18	35.02	55.18	43.15	48.15
	Colorless Pigments (ug/g)	45.28	35.28	55.25	43.25	48.20
Cyanidin glycoside	purplish - red	1.72	18.43	44.63	27.28	43.38
Malvidin glycoside	purplish - red	9.71	272.86	481.79	883.18	1198.11
Pelargonidin glycoside	red	0.82	16.34	44.20	7.14	12.58
Peonidin glycoside	purplish - red	0.32	2.92	7.01	4.03	6.95
Petunidin glycoside	reddish brown	0.43	17.60	21.83	42.34	65.32
	Red pigments (ug/g)	13.00	328.16	599.45	963.97	1326.35
Delphinidin glycoside	blue	9.27	1261.11	1716.66	2747.78	3027.64
carotene	orange-yellow	10.30	5.50	4.68	7.56	9.57
lutein	yellow	126.61	78.77	76.23	90.34	111.27
	Yellow pigments(ug/g)	136.91	84.28	80.91	97.91	120.84
	The proportion between the content of blue pigment and that of red pigment	0.71	3.84	2.86	2.85	2.28

In BH, the content of each color pigment was relatively low: yellow pigment was the most abundant, at 136.91 µg/g, while the content of other colors was less than 10 µg/g.

### 3.3 Transcriptome results

#### 3.3.1 Alignment and annotation of assembled sequences

In this study, a total of 174, 531 genes were assembled and mapped to the reference genome. Of these assembled and mapped genes, 164, 632 genes were successfully annotated with the reference genome. Among the remaining 9, 899 genes, 2, 648 were not annotated in the NR database (**Supplementary File S3**).

#### 3.3.2 CSDEGs and their expression analysis

##### 3.3.2.1 CSDEGs

**Figure 2:**
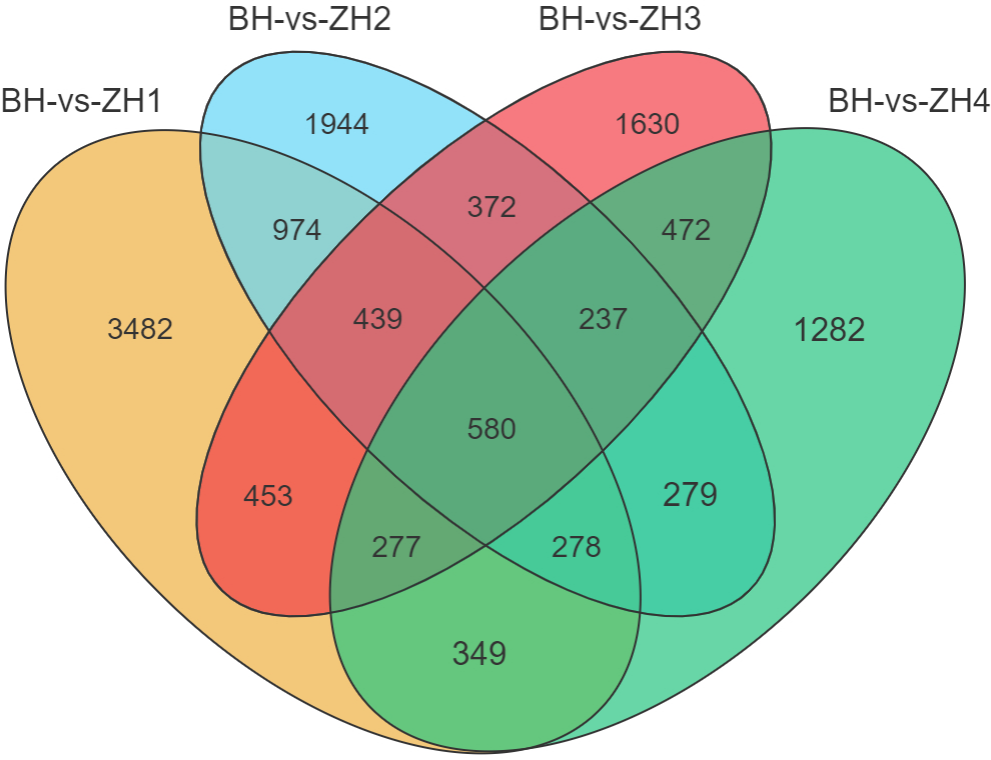
Venn diagram of SDEGs among the four groups.

The SDEGs between BH and ZH1, ZH2, ZH3, and ZH4 are detailed in **Supplementary Files S4**–**S7**, respectively. TSDEGs are detailed in **Supplementary File S8**. A total of 580 CSDEGs were identified between BH and the four ZH groups (ZHs) (**Figure 2**; **Supplementary File S9**). Among these CSDEGs, 497 genes exhibited higher relative expression in ZHs compared to BH, accounting for approximately 85.69%. Conversely, 83 genes had lower relative expression in ZHs than in BH, representing about 14.31%. Notably, 245 CSDEGs were detected in ZHs but not in BH, including the unannotated transcript MSTRG.14861. In contrast, 13 genes were detected in BH but not in ZHs.

Fifty CSDEGs were unannotated in the reference genome. Among them, five genes (MSTRG.14861, MSTRG.59017, MSTRG.74352, MSTRG.76242, MSTRG.104060) were not annotated in NR database, and their nucleotide sequences are provided in detail in **Supplementary File S10**. The relative expression levels of the first four unannotated genes in BH were significantly lower than those in ZHs, while the relative expression of the fifth unannotated gene was significantly higher in BH than in ZHs (**Table 3**).

**Table 3:** Relative expression abudance of five genes not annotated in NR.

	MSTRG.104060	MSTRG.14861	MSTRG.59017	MSTRG.74352	MSTRG.76242
BH	2.77 ± 1.26	0.00 ± 0.00	0.03 ± 0.03	0.38 ± 0.09	7.75 ± 2.00
ZH1	0.00 ± 0.00	7.59 ± 3.97	0.94 ± 0.12	1.79 ± 0.35	52.93 ± 15.23
ZH2	0.00 ± 0.00	8.09 ± 4.07	1.47 ± 0.82	3.64 ± 1.08	98.84 ± 25.23
ZH3	0.00 ± 0.00	9.58 ± 6.85	2.01 ± 1.24	3.4 ± 1.95	44.04 ± 7.88
ZH4	0.00 ± 0.00	8.89 ± 5.94	2.04 ± 0.86	2.71 ± 0.57	76.6 ± 15.71
profile	0	8	8	8	8

##### 3.3.2.2 Trends analysis results of CSDEGs

**Figure 3:**
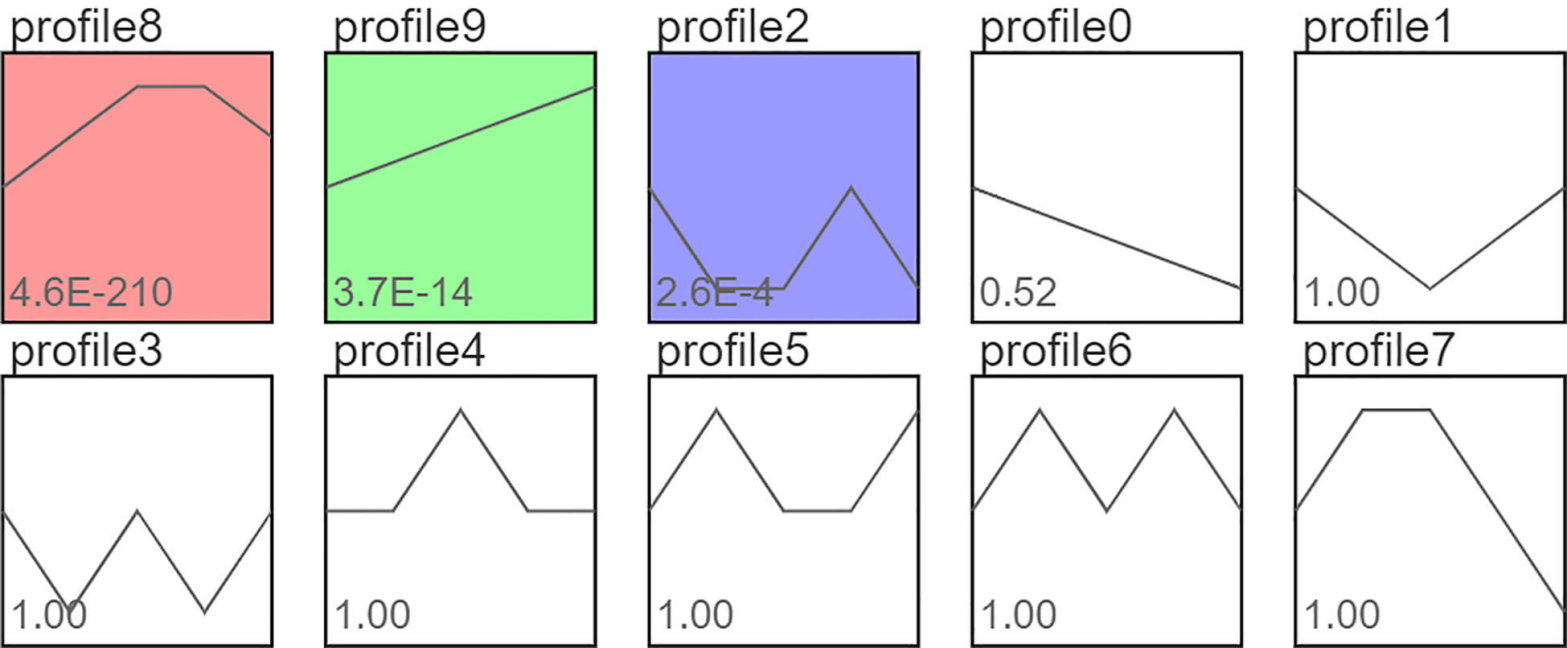
Relative expression trend classes of CSDEGs in BH, ZH1, ZH2, ZH3 and ZH4.

The expression trends of CSDEGs in BH, ZH1, ZH2, ZH3, and ZH4 are detailed in **Figure 3**; **Supplementary File S11**. Among all expression profiles, genes showing the trends of profile 9, profile 0, and profile 8 are of particular interest. Specifically, Expression trend of 78 CSDEGs belongs to profile 9, Expression trend of 415 CSDEGs belongs to profile 8, Expression trend of 30 CSDEGs belongs to profile 0.

Among the 50 unannotated CSDEGs, Expression trend of 13 unannotated CSDEGs in BH, ZH1, ZH2, ZH3 and ZH4 belongs to profile 9, Expression trend of 34 unannotated CSDEGs belongs to profile 8, Expression trend of 2 unannotated CSDEGs belongs to profile 0. Notably, MSTRG.14861, MSTRG.59017, MSTRG.74352, MSTRG.76242, and MSTRG.104060 were classified under profile 8, with their relative expression levels in BH being significantly lower than those in ZHs. In contrast, MSTRG.104060, which belongs to profile 0, exhibited significantly higher relative expression in BH than in ZHs (**Table 3**).

##### 3.3.2.3 Results of statistical analysis of transcription factors in CSDEGs

Out of the 174, 531 genes, 8, 794 were identified as transcription factors (**Supplementary File S12**). Among the 24 transcription factors in CSDEGs (**Supplementary File S11**), the two most abundant types are MYB transcription factors and bHLH transcription factors, while no WD40 transcription factors were detected. Specifically, the relative expression levels of MS.gene053010 (myb-related protein 305), MSTRG.86353 (MYB-like transcription factor EOBII), MS.gene54772 (Myb DNA-binding domain protein), MS.gene025894 (transcription factor bHLH13), MS.gene033132 (BHLH transcription factor), and MS.gene059664 (transcription factor bHLH18) were significantly higher in ZHs compared to BH. Notably, CSDEGs in profile 9 included myb-related protein 305(MS.gene053010) and MYB-like transcription factor EOBII (MSTRG.86353).

#### 3.3.3 Pathway and GO enrichment analysis of CSDEGs

Pathway and GO Enrichment Analysis result of CSDEGs are detailed in **Supplementary Files S13**, **S14**. There were 458 CSDEGs with enrichment annotation in either KEGG or GO databases. In contrast, there were 122 CSDEGs without any enrichment annotation in either KEGG or GO databases, and a preliminary search into the functions of these 122 genes revealed that the functions of some remain unclear, while many of those with known functions are associated with basic biological processes.

##### 3.3.3.1 Pathway enrichment analysis results of CSDEGs

**Figure 4:**
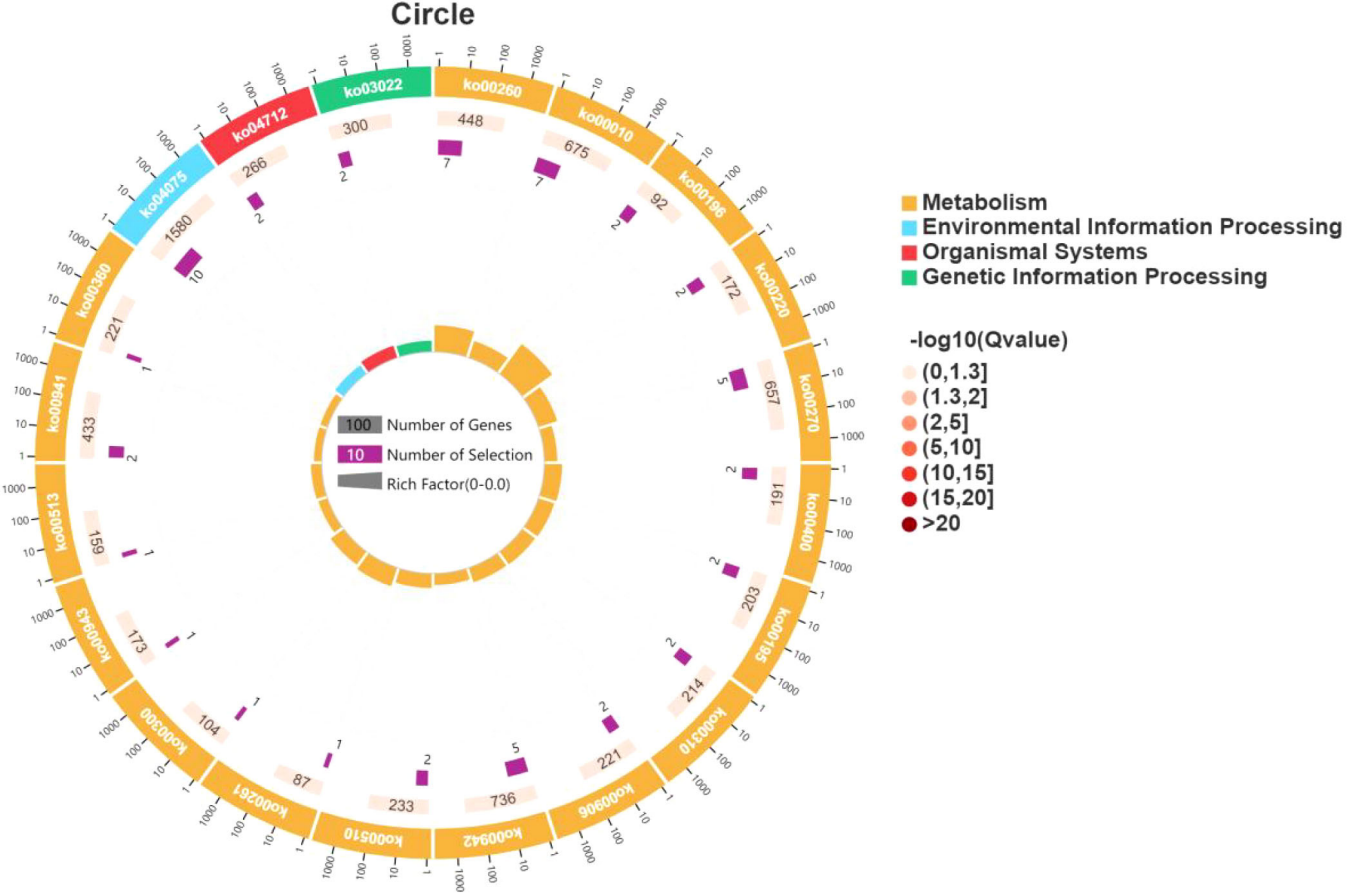
Pathways enriched in CSDEGs.

KEGG analysis revealed that CSDEGs were primarily enriched in the following categories: metabolism, genetic information processing, environmental information processing, cellular process, and organismal systems. The metabolism category included carbohydrate metabolism, amino acid metabolism, lipid metabolism, energy metabolism, secondary metabolite metabolism, terpenoid and polyketide metabolism, nucleotide metabolism, cofactor and vitamin metabolism, and glycan biosynthesis and metabolism. The genetic information processing category included folding, sorting and degradation, translation, transcription, and replication and repair. The environmental information processing category included signal transduction. The cellular process category included transport and catabolism. The organismal systems included environmental adaptation (**Figure 4**).

##### 3.3.3.2 GO enrichment analysis results of CSDEGs

**Figure 5:**
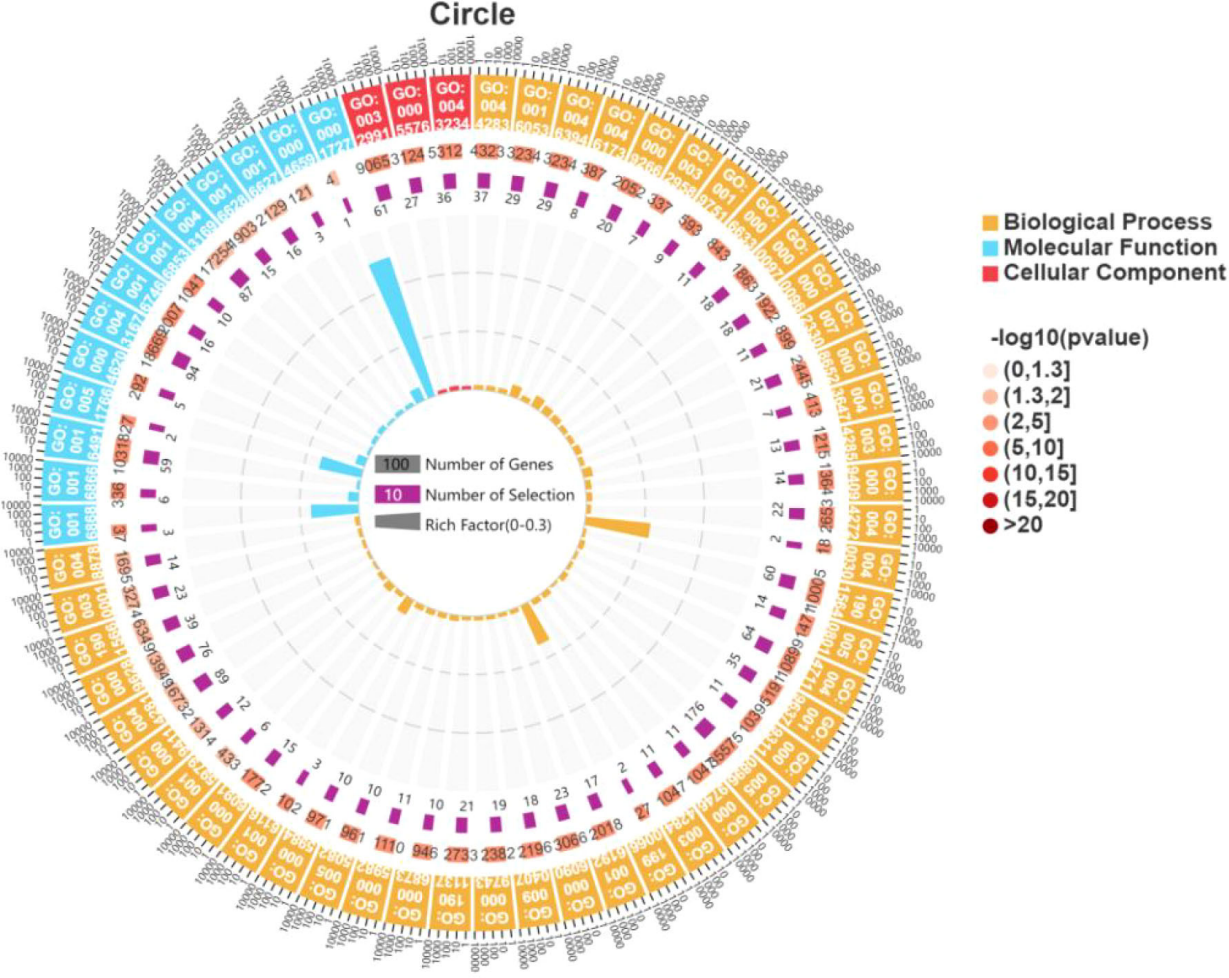
GO terms enriched in CSDEGs.

Significant enrichmen (**Figure 5**) was observed in the following cellular component GO terms: macromolecular complex, protein complex, intracellular ribonucleoprotein complex, proteasome complex, DNA packaging complex, extracellular region, extracellular region part, chloroplast, chloroplast part, and organelle subcompartment.

Significant enrichment was also observed in the following molecular function GO terms: transferase activity, ion binding, oxidoreductase activity, hydrolase activity, phospholipase activity, alpha-1, 3-glucosidase activity, beta-fructofuranosidase activity, mannosyl-oligosaccharide mannosidase activity, isomerase activity, inositol trisphosphate kinase activity, lipid kinase activity, and manganese ion transmembrane transporter activity. Among the genes enriched in transferase activity, there are 27 genes that have transferase function but no clear associated metabolic pathway, of which 9 are glycosyltransferase genes (**Table 4**).

**Table 4:** Genes with transferase function in CSDEGs without KO annotation.

NO.	ID	Symbol	Description
1	MS.gene23161	F26G	beta-glucosidase 12 [Medicago truncatula]
2	MS.gene88922	——	Sterol 3-beta-glucosyltransferase UGT80B1 isoform B [Glycine soja]
3	MS.gene018884	STL1	probable glycosyltransferase STELLO1 [Medicago truncatula]
4	MS.gene033758	XTHB	probable xyloglucan endotransglucosylase/hydrolase protein B [Medicago truncatula]
5	MS.gene070682	OFUT39	O-fucosyltransferase 39 [Medicago truncatula]
6	MS.gene26273	--	nucleotidyltransferase [Medicago truncatula]
7	MS.gene62899	PGSIP8	Glycogenin-2 [Glycine soja]
8	MSTRG.53878	At4g19900	nucleolar complex protein 2 homolog [Medicago truncatula]
9	MSTRG.88570	XTH1	xyloglucan endotransglycosylase [Medicago truncatula]
10	MS.gene63944	At3g02910	gamma-glutamylcyclotransferase [Trifolium pratense]
11	MS.gene00866	VIT_05s0020g02800	ATP-dependent (S)-NAD(P)H-hydrate dehydratase isoform X3 [Medicago truncatula]
12	MS.gene013090	RGTA1	geranylgeranyl transferase type-2 subunit alpha 1 [Medicago truncatula]
13	MS.gene023804	PFC1	ribosomal RNA small subunit methyltransferase, chloroplastic [Medicago truncatula]
14	MS.gene037432	DCR	uncharacterized acetyltransferase At3g50280 [Medicago truncatula]
15	MS.gene052358	NMT1	glycylpeptide N-tetradecanoyltransferase 1 isoform X2 [Medicago truncatula]
16	MS.gene056288	PAT24	DHHC-type zinc finger protein [Medicago truncatula]
17	MS.gene05804	SCPL12	serine carboxypeptidase-like 12 [Medicago truncatula]
18	MS.gene071814	FTIP3	FT-interacting protein 1 [Medicago truncatula]
19	MS.gene073461	EEF1AKN	MT EEF1A lysine methyltransferase 4 [Medicago truncatula]
20	MS.gene22903	HSR201	benzyl alcohol O-benzoyltransferase [Medicago truncatula]
21	MS.gene31322	SIZ1	E3 SUMO-protein ligase SIZ1 [Medicago truncatula]
22	MS.gene83461	BEAT	omega-hydroxypalmitate O-feruloyl transferase [Medicago truncatula]
23	MSTRG.49897	EPS1	HXXXD-type acyl-transferase family protein [Medicago truncatula]
24	MS.gene26675	Pmr5/Cas1p	GDSL/SGNH-like acyl-esterase family protein [Medicago truncatula]
25	MS.gene64828	STR15	thiosulfate sulfurtransferase 16, chloroplastic [Medicago truncatula]
26	MS.gene44392	JMJ25	transcription factor jumonji (JmjC) domain protein [Medicago truncatula]
27	MS.gene47747	ROPGAP7	GTPase-activator protein for Rho-like GTPase family protein [Medicago truncatula]

Significant enrichment was observed in the following biological process GO terms: metabolic and biosynthetic process (carboxylic acid metabolic and biosynthetic process, cellular amino acid metabolic and biosynthetic process, organophosphate metabolic and biosynthetic process, Secondary metabolite metabolic and biosynthetic process, metal ion, auxin, Golgi vesicle, photosynthetic electron transport, pigment accumulation in tissues in response to UV light, etc), response to abiotic stimulus (response to radiation, response to temperature stimulus, response to water), response to endogenous stimulus, response to chemical (response to organic substance, response to carbohydrate, response to disaccharide, response to hexose, response to monosaccharide), response to inorganic substance). homeostatic process (ion homeostasis, chemical homeostasis), system development (reproductive structure development, endosperm development).

##### 3.3.3.3 Metabolic pathways and CSDEGs involved in anthocyanin glycoside synthesis chain and their expression trends

Based on KEGG data, the anthocyanin biosynthesis chain was derived backward from the anthocyanin biosynthesis pathway. This chain includes the following pathways: photosynthesis, glycolysis/gluconeogenesis, glycine, serine, and threonine metabolism, monobactam biosynthesis, cysteine and methionine metabolism, lysine biosynthesis, arginine and proline metabolism, pentose phosphate pathway, phenylalanine, tyrosine, and tryptophan biosynthesis, phenylpropanoid biosynthesis, flavonoid biosynthesis, and anthocyanin biosynthesis. Notably, only 29 genes were definitively involved in these pathways, and the significance levels of these KEGGs enriched by CSDEGs were not high (**Table 5**).

**Table 5:** Metabolic pathways and CSDEGs involved in anthocyanin glycoside synthesis chain.

Pathway//ko	K_ID	ID	Description	Profile	up/down
Photosynthesis//ko00195	K02636	MS.gene029004	cytochrome b6-f complex iron-sulfur subunit, chloroplastic [Medicago truncatula]	8	up
	K02115	MS.gene027054	ATP synthase gamma chain 2, chloroplastic [Medicago truncatula]	8	up
Photosynthesis — antenna proteins//ko00196	K08909	MS.gene017490	chlorophyll a-b binding protein 3, chloroplastic [Medicago truncatula]	7	up
	K08909	MS.gene071930	chlorophyll a-b binding protein CP24 10A, chloroplastic [Medicago truncatula]	8	up
Glycolysis/Gluconeogenesis//ko00010	K00873	MS.gene005813	pyruvate kinase 1, cytosolic [Medicago truncatula]	8	up
	K03841	MS.gene03323	fructose-1, 6-bisphosphatase, cytosolic [Medicago truncatula]	0	down
	K15633	MS.gene036906	2, 3-bisphosphoglycerate-independent phosphoglycerate mutase [Medicago truncatula]	9	up
	K00873	MS.gene20006	pyruvate kinase 1, cytosolic [Medicago truncatula]	8	up
	K15633	MS.gene27538	2, 3-bisphosphoglycerate-independent phosphoglycerate mutase [Medicago truncatula]	9	up
	K15633	MS.gene27540	2, 3-bisphosphoglycerate-independent phosphoglycerate mutase [Medicago truncatula]	9	up
	K00927	MS.gene74184	phosphoglycerate kinase 3, cytosolic [Medicago truncatula]	8	up
Glycine, serine and threonine metabolism//ko00260	K00831	MS.gene000463	phosphoserine aminotransferase 2, chloroplastic [Medicago truncatula]		up
	K15633	MS.gene036906	2, 3-bisphosphoglycerate-independent phosphoglycerate mutase [Medicago truncatula]	9	up
	K15633	MS.gene27538	2, 3-bisphosphoglycerate-independent phosphoglycerate mutase [Medicago truncatula]	9	up
	K15633	MS.gene27540	2, 3-bisphosphoglycerate-independent phosphoglycerate mutase [Medicago truncatula]		up
	K14272	MS.gene44305	glutamate—glyoxylate aminotransferase 2 [Medicago truncatula]		up
	K00276	MS.gene69728	primary amine oxidase [Medicago truncatula]		up
	K12524	MS.gene75658	bifunctional aspartokinase/homoserine dehydrogenase 2, chloroplastic isoform X1 [Medicago truncatula]		up
Monobactam biosynthesis//ko00261	K12524	MS.gene75658	bifunctional aspartokinase/homoserine dehydrogenase 2, chloroplastic isoform X1 [Medicago truncatula]		up
Cysteine and methionine metabolism//ko00270	K00831	MS.gene000463	phosphoserine aminotransferase 2, chloroplastic [Medicago truncatula]		up
	K00547	MS.gene006428	homocysteine S-methyltransferase-like protein [Medicago truncatula]		down
	K05933	MS.gene05789	1-aminocyclopropane-1-carboxylate oxidase [Medicago truncatula]		up
	K00640	MS.gene66256	serine acetyltransferase 1, chloroplastic [Medicago truncatula]		up
	K12524	MS.gene75658	bifunctional aspartokinase/homoserine dehydrogenase 2, chloroplastic isoform X1 [Medicago truncatula]		up
Lysine biosynthesis//ko00330	K12524	MS.gene75658	bifunctional aspartokinase/homoserine dehydrogenase 2, chloroplastic isoform X1 [Medicago truncatula]		up
Arginine and proline metabolism//ko00330	K17839	MS.gene019144	probable polyamine oxidase 2 [Medicago truncatula]		up
Pentose phosphate pathway//ko00030	K03841	MS.gene03323	fructose-1, 6-bisphosphatase, cytosolic [Medicago truncatula]	0	down
Phenylalanine, tyrosine and tryptophan biosynthesis//ko00400	K05359	MS.gene049847	arogenate dehydratase/prephenate dehydratase 2, chloroplastic [Medicago truncatula]	2	down
	K05359	MS.gene049849	arogenate dehydratase/prephenate dehydratase 2, chloroplastic [Medicago truncatula]	2	down
Phenylpropanoid biosynthesis//ko00940	K00430	MS.gene02084	peroxidase 15 isoform X1 [Medicago truncatula]	8	up
Flavonoid biosynthesis//ko00941	K00660	MS.gene033730	chalcone synthase [Medicago truncatula]	8	up
	K08243	MSTRG.76939	NAD(P)H-dependent 6’-deoxychalcone synthase-like [Cicer arietinum]	9	up
Anthocyanin biosynthesis//ko00942	K12930	MS.gene069129	7-deoxyloganetic acid glucosyltransferase isoform X1 [Medicago truncatula]	8	up
	K12338	MS.gene71667	gallate 1-beta-glucosyltransferase [Medicago truncatula]	8	up
	K17193	MS.gene89382	soyasaponin III rhamnosyltransferase [Medicago truncatula]	9	up
	K21383	MS.gene91596	phenolic glucoside malonyltransferase 1 [Medicago truncatula]	9	up
	K213833	MS.gene91614	phenolic glucoside malonyltransferase 1 [Medicago truncatula]	8	up

As shown in **Table 5**, only the relative expression levels of MS.gene03323 (FBPban1), MS.gene049847 (ADT2), and MS.gene049849 (ADT2) in BH were significantly higher than those in ZH1, ZH2, ZH3, and ZH4. The relative expression levels of the other genes in BH were significantly lower than those in ZH1, ZH2, ZH3, and ZH4; specifically, the relative expression of these CSDEGs belonging to profile 9 increased progressively in the order of BH, ZH1, ZH2, ZH3, and ZH4.

##### 3.3.3.4 Analysis result of CSDEGs not enriched in KEGG pathways

Thirty four important genes from CSDEGs not enriched in KEGG pathways were chosen (**Supplementary File S15**), and these genes are potentially involved in flower color formation. They are classified into four gene categories (key enzymes for pigment biosynthesis and modification, genes involved in pigment transport and vacuolar compartmentalization, core transcription regulatory factors, genes involved in signal and modification, genes involved in RNA regulation and protein modification and degradation).

##### 3.3.3.5 qRT-PCR result

**Figure 6:**
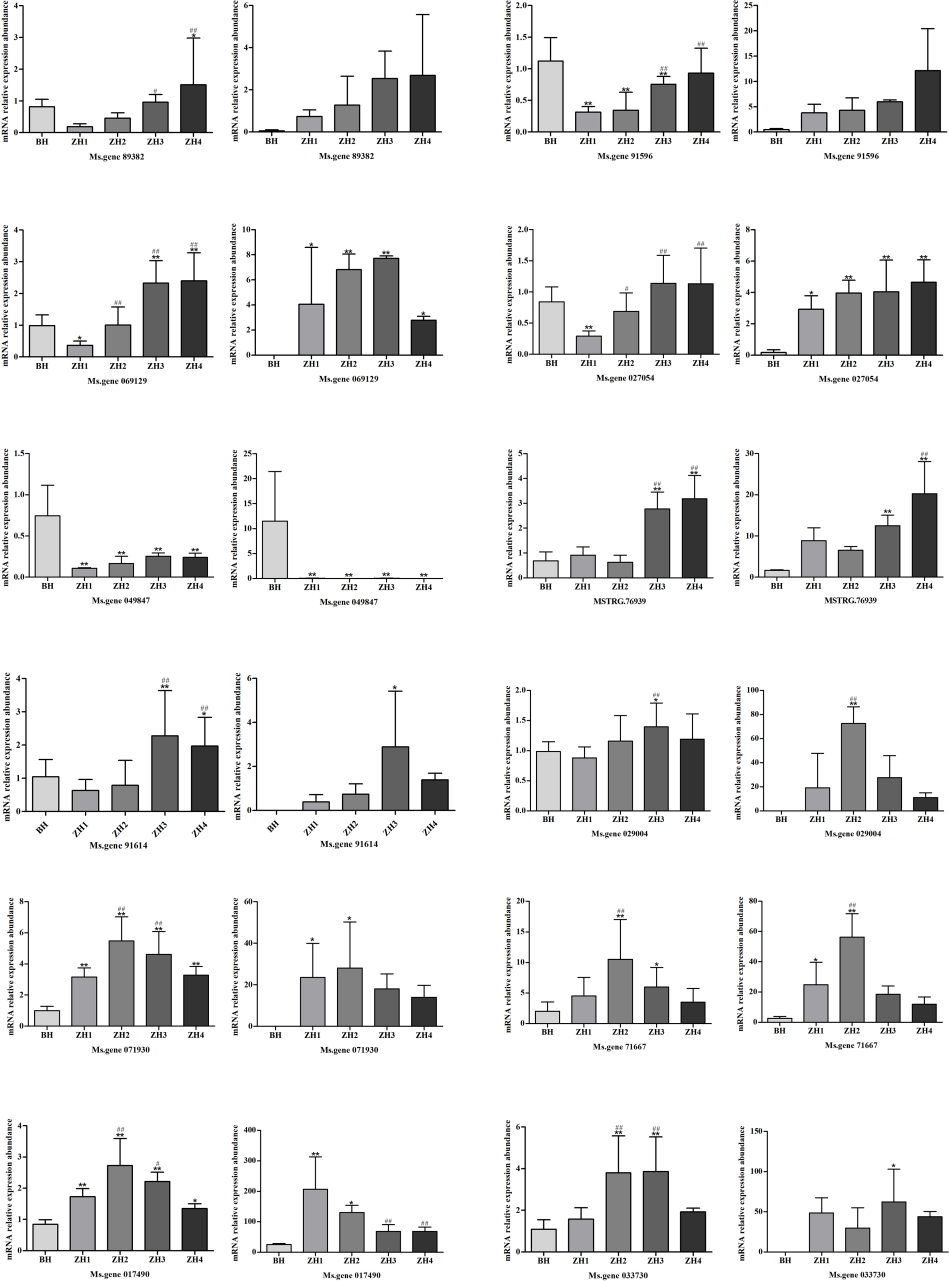
mRNA relative expression abundance of these gene in BH, ZH1, ZH2, ZH3, and ZH4 by qRT-PCR and transcriptome data Note: Left panel: qRT-PCR data, right panel: transcriptome data for the same gene. The mRNA content difference between ZH1, ZH2, ZH3, ZH4 and BH is marked with *, The mRNA content difference betweenZH2, ZH3, ZH4 and ZH1 is marked with #. (^*#&^P < 0.05; ^**##&&^P < 0.01). Error bars indicate standard deviation (SD).

Among the 12 selected genes, the fluorescence quantitative results of 4 genes including Ms.gene071930, Ms.gene049847, Ms.gene71667 and Ms.gene76939 showed a completely consistent trend with the transcriptome results. The fluorescence quantitative results of the remaining 8 genes including Ms.gene017490, Ms.gene027054, Ms.gene89382, Ms.gene91614, Ms.gene91596, Ms.gene033730, Ms.gene069129 and Ms.gene029004 showed a basically consistent trend with the transcriptome results (**Figure 6**). These results confirm that the RNA-seq expression data are authentic and reliable.

### 3.4 Results of combined transcriptome and metabolome analysis

Only 17 of the 64 anthocyanins were found in the anthocyanin synthesis pathway, and 12 of them detected in the samples(**Supplementary File S1**). The CSDEGs involved in their synthesis were only MS.gene069129 (BZ1) and MS.gene71667 (UGT75C1) (**Table 6**). Notably, the relative expression levels of MS.gene069129 (BZ1) and MS.gene71667 (UGT75C1) in ZHs were significantly higher than those in BH. Furthermore, except for pelargonidin-3-O-(6-O-malonyl-beta-D-glucoside), the content of the other 11 anthocyanins in ZHs was significantly higher than that in BH (**Supplementary File S1**).

**Table 6:** Anthocyanin glycosides in anthocyanin synthesis pathway and CSDEGs involved in its synthesis.

Compounds	CSDEGs
Cyanidin-3-O-glucoside	MS.gene069129(K12930 BZ1)
Cyanidin-3, 5-O-diglucoside	MS.gene069129 (K12930 BZ1), MS.gene71667(K12338 UGT75C1)
Cyanidin-3-O-(6-O-malonyl-beta-D-glucoside)	MS.gene069129 (K12930 BZ1)
Peonidin-3-O-glucoside	MS.gene069129 (K12930 BZ1)
Delphinidin-3-O-glucoside	MS.gene069129 (K12930 BZ1)
Delphinidin-3, 5-O-diglucoside	MS.gene069129 (K12930 BZ1), MS.gene71667(K12338 UGT75C1)
Delphinidin-3-O-(6-O-malonyl)-glucoside-3’-glucoside	MS.gene069129 (K12930 BZ1)
Petunidin-3-O-glucoside	MS.gene069129 (K12930 BZ1)
Malvidin-3-O-glucoside	MS.gene069129 (K12930 BZ1)
Pelargonidin-3-O-glucoside	MS.gene069129 (K12930 BZ1)
Pelargonidin-3, 5-O-diglucoside	MS.gene069129 (K12930 BZ1), MS.gene71667(K12338 UGT75C1)
Pelargonidin-3-O-(6-O-malonyl-beta-D-glucoside)	MS.gene069129 (K12930 BZ1)

Correlation analysis identified 128 CSDEGs whose relative expression levels exhibited strong and significant correlations with delphinidin-3, 5-O-diglucoside content across BH, ZH1, ZH2, ZH3, and ZH4 (**Supplementary File S16**). Likewise, 84 CSDEGs were strongly and significantly associated with malvidin-3, 5-O-diglucoside content in the same accessions (**Supplementary File S17**). Notably, the unannotated transcript MSTRG.59017 showed a pronounced positive correlation with both anthocyanins, whereas MSTRG.14861 was selectively and highly correlated with delphinidin-3, 5-O-diglucoside (**Table 7**).

**Table 7:** Unannotated genes of significant correlation with Delphinidin-3, 5-O-diglucoside, Malvidin-3, 5-O-diglucoside.

Transcript id	Profile	BH	ZH1	ZH2	ZH3	ZH4	Anthocyanin glycosides	Correlation	p_value
MSTRG.59017	8	0	5.14	5.79	6.24	6.25	Delphinidin-3, 5-O-diglucoside	0.9881	0.0016
MSTRG.59017	8	0	5.14	5.79	6.24	6.25	Malvidin-3, 5-O-diglucoside	0.9318	0.0212
MSTRG.14861	8	0	12.89	12.98	13.23	13.12	Delphinidin-3, 5-O-diglucoside	0.8884	0.0440

## 4 Discussion

In the study, metabolomic analysis revealed that the content of anthocyanins is far higher than that of carotenoids. Specifically, the content of anthocyanins in purple flowers is much higher than that in white flowers, whereas the content of carotenoids in white flowers is higher than that in purple flowers (**Supplementary Files S1**, **S2**). This finding indicates that the pigment responsible for the purple color of the flowers is anthocyanin rather than carotenoid. Furthermore, the contents of various detected anthocyanins in purple flowers were significantly higher than those in white flowers, and showed an increasing trend from white flowers, weak light purple, light purple, purple to deep purple. The content ratio of blue and red anthocyanins gradually decreased from the white-to-deep-purple color series (**Table 2**). Among these anthocyanins, delphinidin and cyanidin were the main color-forming anthocyanins, whose contents accounted for an absolute advantage in the total pigment content. The transcriptomic analysis obtained important genes strongly associated with flower color formation, and the relative expression levels of these genes showed a regular trend from the white-to-deep-purple color series (**Supplementary File S11**). Most of which are significantly involved in external environment, internal environment, signal transduction, transcriptional regulation (especially transcription factors), translational regulation, as well as the synthesis and transport of anthocyanins (**Supplementary Files S13**, **S14**). Combined transcriptomic and metabolomic analysis revealed that the only BZ1(MS.gene069129) and UGT75C1(MS.gene71667) are involved in the synthesis of anthocyanins. Specifically, the unannotated MSTRG.59017 and MSTRG.14861 regulate positively synthesis of delphinidin-3, 5-O-diglucoside, and the unannotated MSTRG.59017 also regulate positively synthesis of malvidin-3, 5-O-diglucoside (**Table 7**). These discoveries have enriched the knowledge regarding the mechanism of plant flower color formation, and have also preliminarily clarified the mechanism underlying the formation of varying shades of purple in alfalfa purple flowers.

### 4.1 The metabolism of three major substances serves as the material basis for pigment synthesis

The KEGG pathway enrichment analysis showed that pathways related to carbohydrate metabolism (such as glycolysis/gluconeogenesis) and lipid metabolism (such as terpenoid backbone biosynthesis and carotenoid biosynthesis) were significantly enriched. Among these pathways, glycolysis and the pentose phosphate pathway provide phenylpropanoid precursors (such as 4-coumaroyl-CoA) for anthocyanin synthesis; lipid metabolism genes (such as those related to phospholipase activity) may be involved in membrane transport or signal transduction. This result supports previous theories that metabolic networks underpin the synthesis of secondary metabolites.

### 4.2 Genes responding to the intracellular and extracellular environment influencing the purple formation of alfalfa flowers

Previous studies have clarified that the color of pigments is influenced by external environmental factors (e.g., light, temperature, water, soil nutrients, and organisms) and internal environmental factors (e.g., sugar, metal ions, pH value), focusing on how environmental changes affect flower color (Zhao et al., 2005; Tanaka et al., 2008; Yoshida, 2024). However, this study investigated the formation mechanism of different flower colors under consistent external environmental conditions. CSDEGs responding to external stimuli (such as light, temperature, and water) were highly expressed in purple flowers (**Supplementary Files S9**, **S14**) and it is hypothesized that they may indirectly regulate anthocyanin synthesis by modulating other genes (e.g. transcription factors). Therefore, under the same environmental conditions, the distinct responses of floral organs (cells) with different flower colors to external environments constitute the genetic basis and critical cause for the formation of different flower colors.

Intracellular chemical stimulation also plays a crucial role in flower color formation (Zhao and Tao, 2015). Transcriptome analysis revealed that CSDEGs were significantly enriched in “response to endogenous stimulus” and “response to chemical” (including response to disaccharide, response to hexose, response to monosaccharide, and response to inorganic substance) (**Supplementary File S14**), which is consistent with previous studies. This indicates that flowers with different colors exhibit distinct internal environments and responses to endogenous sugars and metal ions. These findings may be closely associated with changes in the extracellular environment, echoing previous research on how environmental factors influence plant physiological and biochemical processes. Although this study did not directly explore how these chemicals stimulate flower color formation, their presence and variations undoubtedly provide the necessary basis for flower color development.

KEGG analysis showed that 10 CSDEGs involved in signal transduction (environmental information processing) (**Supplementary File S13**). Specifically, the expression levels of serine/threonine-protein kinase SRK2I (SAPK7, 3), BRASSINOSTEROID INSENSITIVE 1-associated receptor kinase 1, auxin transporter-like protein 3, cyclin-D3–2 and probable xyloglucan endotransglucosylase/hydrolase protein 23 in ZHs are significantly more than that in BH; whereas the expression levels of auxin-responsive protein SAUR32 in ZHs is significantly lower than that in BH. Thus, they are likely to be involved in flower color formation. However, there are currently no direct research results supporting their involvement in flower color formation.

### 4.3 Regulation genes for transcriptional, translational and post-translational of anthocyanin biosynthesis

Floral anthocyanin biosynthesis largely depends on the conserved MYB-bHLH-WD40 (MBW) activation complex, with MYB repressors acting hierarchically with the MBW complex (Li et al., 2020). Transcription factors (TFs) of the MYB and bHLH families play a vital role in the regulation of anthocyanin biosynthesis (Schwinn et al., 2006; Zhou et al., 2024; Wang et al., 2019). We identified 49 MYBs and 24 bHLHs in the comparison of purple flowers vs. cream flowers (**Supplementary File S12**). suggesting that these MYB and bHLH TFs may also be involved in regulating the formation of alfalfa flower color. Previous studies have shown that myb-related protein 305 (MS.gene053010) may directly activate the expression of anthocyanin synthesis genes in alfalfa (Borevitz et al., 2000; Gonzalez et al., 2008; Zhang et al., 2023). MYB-like transcription factor EOBII regulates the biosynthesis of flower fragrance volatiles (Spitzer-Rimon et al., 2010), and probably shares a co-regulatory network with the flower color pathway (Cna'ani et al., 2015). Notably, a novel finding of this study is that MYB-like transcription factor EOBII (MSTRG.86353) was only the transcription factor showing an increasing trend in BH, ZH1, ZH2, ZH3, and ZH4, implying its potential involvement in regulating alfalfa flower color formation. Additionally, transcription factor bHLH13 might promote anthocyanin accumulation through positive regulation of SmCHS and SmF3H by forming the MBW complex together with MYB and WD40 (Xi et al., 2021).

WRKY TFs responsible for regulating anthocyanin biosynthesis have been reported (Liu et al., 2019). We identified 27 WRKY TFs in the comparison of purple flowers vs. cream flowers(**Supplementary File S12**). Therefore, they are most likely regulating anthocyanin biosynthesis in alfalfa. A recent study has demonstrated that WRKY transcription factor 21 affects flower color by binding to W-box and regulating the metabolism of anthocyanins (Guo et al., 2025).

Studies have shown that ethylene-responsive transcription factor ERF106 (ERF106) may compete for binding to bHLH factors to inhibit the formation of the MBW complex, reduce anthocyanin accumulation, lighten flower color (toward yellow/orange hues), and affect the pigmentation of fruits and floral organs. It alters the ratio of anthocyanins to volatiles, thereby indirectly affecting the intensity of flower color by influencing the distribution of phenylpropanoid metabolic flux (Min et al., 2012; Zhang et al., 2018; Yin et al., 2024; Wang et al., 2023). This study indicate that ERF106 content in the purple-flowered group was much lower than that in the white-flowered group, suggesting that the down-regulation of ERF106 is conducive to the accumulation of anthocyanins - a finding consistent with previous studies. Ethylene-responsive transcription factor ERF017 (ERF107) may respond to ethylene signals to regulate flower color (Wang et al., 2023). This study indicate that ERF107 content in the purple-flowered group was much higher than that in the white-flowered group, suggesting that the up-regulation of ERF107 is conducive to the accumulation of anthocyanins. A study has demonstrated that BZIP transcription factor may play a positive role in anthocyanin accumulation (Zhang et al., 2025). Consistently, this study indicated that its content in the purple-flowered group was higher than that in the white-flowered group, implying that its up-regulation promotes anthocyanin accumulation.

F-box proteins play an important role for translational and post-translational network in anthocyanin biosynthesis. F-box genes downregulation increases the content of certain flavonoids, resulting in deep purple flower color (Liu et al., 2023; Jo et al., 2020). However, this study indicated that F-box genes content in the purple-flowered group was higher than that in the white-flowered group, suggesting that F-box genes up-regulation increases the accumulation of anthocyanins - a result that contradicts both aforementioned studies. This opposite regulatory pattern may be attributed to species-specific, tissue-specific regulation. Protein argonaute 5 affects the expression level of flavonoid synthases by regulating the mRNA stability and translation efficiency of flower color-related genes (Mach, 2017). This study indicated that protein argonaute 5 content in the purple-flowered group was higher than that in the white-flowered group, suggesting that it up-regulation increases the accumulation of anthocyanin.

### 4.4 Synthesis of anthocyanins

Anthocyanins are the main pigment components in plant flowers and play a decisive role in the formation of flower colors (Grotewold, 2006). Among the 29 CSDEGs definitively involved in the anthocyanin biosynthesis chain, 25 exhibited significantly higher relative expression levels in ZHs than in BH (**Table 5**), and their high expression led to higher contents of pelargonidin glycosides, cyanidin glycosides, and delphinidin glycosides in ZHs compared to BH. For example, chalcone synthase (CHS) is the first key enzyme in the synthesis of Pelargonidin, Cyanidin, and Delphinidin in the flavonoid biosynthesis pathway. Notably, CHS(MS.gene033730) is significantly highly expressed in purple flowers, resulting in a higher content of proanthocyanidins synthesized in purple flowers compared to white flowers. BZ1(MS.gene069129), UGT75C1(MS.gene71667), UGT79B1 (MS.gene89382), and 3AT (MS.gene91596 and MS.gene91614) are involved in the anthocyanin biosynthesis pathway. Specifically, BZ1 acts as an enzyme for synthesizing anthocyanin 3-glucoside, and UGT75C1 primarily functions as an enzyme for synthesizing anthocyanin 3, 5-diglucoside. Their high expression in purple flowers contributes to significantly higher anthocyanin contents in purple flowers compared to white flowers.

Analysis result of CSDEGs not enriched in KEGG pathways or GO terms show that cytochrome P450 family protein, coumaroyl-CoA:anthocyanidin 3-O-glucoside-6’’-O-coumaroyltransferase 1, HXXXD-type acyl-transferase family protein, and 4-coumarate--CoA ligase-like 6 may play important role in anthocyanins biosynthesis and modification (**Supplementary File S16**). Cytochrome P450 family protein may affect the pigment structure and flower color by participating in the hydroxylation or cyclization modification of flavonoids (e.g., anthocyanins) (Tanaka and Brugliera, 2013). Coumaroyl-CoA:anthocyanidin 3-O-glucoside-6’’-O-coumaroyltransferase 1 is involved in the acylation of the 6’’ position of the 3-O-glucose residue of anthocyanin, and also able to use flavonol 3-glucosides as the acyl acceptor, it acts as a flower color stability enhancement factor that determines hue transition, enhances pigment stability, and contributes to the development of bluish hues (Yamazaki et al., 2002; Suzuki et al., 2002).

According to the existing information on the flavonoid biosynthesis pathway, as long as flavonoid 3’, 5’-hydroxylase (EC 1.14.14.81) is present, the content of delphinidin in any plant organ or tissue must be higher than that of cyanidin, and the content of cyanidin must be higher than that of pelargonidin, and the current anthocyanin biosynthesis map indicates that the enzymes responsible for synthesizing the glycosides of delphinidin, cyanidin, and pelargonidin are identical, meaning the content of delphinidin glycosides must be higher than that of cyanidin glycosides, which in turn must be higher than that of pelargonidin glycosides. Therefore, this suggests that the contents of delphinidin, cyanidin, and pelargonidin glycosides are determined by flavonoid biosynthesis.

In anthocyanin biosynthesis, dihydroquercetin is catalyzed by dihydroflavonol 4-reductase (EC 1.1.1.219) and anthocyanidin synthase (EC 1.14.20.4) to converted to cyanidin, and dihydroquercetin is catalyzed by flavonoid 3’, 5’-hydroxylase (EC 1.14.14.81), dihydroflavonol 4-reductase (EC 1.1.1.219) and anthocyanidin synthase (EC 1.14.20.4) to converted to delphinidin. Our transcriptome results showed that the relative expression level of flavonoid 3’, 5’-hydroxylase (EC 1.14.14.81) genes was much higher than that of dihydroflavonol 4-reductase (EC 1.1.1.219), which is the main reason why delphinidin glycosides contents were far higher than cyanidin glycosides contents in the metabolome.

The order of anthocyanin glycosylation is well established in most plant species: glucosylation at the C3 position takes place first, followed by further glucosylation at the C5 position by anthocyanidin 5-O-glucosyltransferase (5GT) to form anthocyanidin 3, 5-O-diglucosides. However, not all plant species possess active 5GT (Springob et al., 2003; Tanaka et al., 2008). In this study, the contents of delphinidin-3, 5-diglucoside and malvidin-3, 5-diglucoside were significantly higher than those of delphinidin-3-glucoside and malvidin-3-glucoside, respectively, which confirms this conclusion. This indicates that most, but not all, of delphinidin-3-glucoside and malvidin-3-glucoside are further glycosylated at the C5 hydroxyl group to form the corresponding 3, 5-diglucosides and alfalfa possesess active 5GT.

The existing anthocyanin biosynthesis pathway shows that the key enzymes involved in the synthesis of multiple glycosides of pelargonidin, delphinidin, and cyanidin in the anthocyanin biosynthesis pathway are shared. Nevertheless, our anthocyanin glycoside metabolomic data revealed that the content order of the corresponding glycosides of delphinidin, cyanidin and pelargonidin does not consistently follow the pattern of delphinidin > cyanidin > pelargonidin consistently across different samples. Furthermore, there are significant differences in the content rankings of distinct delphinidin glycosides, cyanidin glycosides, and pelargonidin glycosides among different samples (**Supplementary File S1**). These results indicate that the pigment glycoside synthesis pathway is incomplete, there may be key genes specific to the synthesis of each anthocyanin glycoside, although their identities remain unclear, and which will be our future research direction. In the study, CSDEGs with transferase function but without ko annotationmay be important candidate genes for improving and enriching anthocyanin biosynthesis pathway, especially the 9 glycosyltransferase genes (**Table 4**).

In addition, correlation analysis revealed that the unannotated MSTRG.59017 is probably involved in synthesis of both delphinidin-3, 5-O-diglucoside and malvidin-3, 5-O-diglucoside, and the unannotated MSTRG.14861 likely also participates in synthesis of delphinidin-3, 5-O-diglucoside (**Table 7**).

### 4.5 Anthocyanin transport and deposition

Petals final color relies on the efficient transport and accumulation of pigments in epidermal cell vacuoles. This study found that several CSDEGs were significantly enriched in pigment transport and accumulation (**Supplementary File S13**). Previous studies have shown that heavy metal-associated isoprenylated plant protein 6 (HIPP6) may act as a glutathione S-transferase chaperone to participate in the transport of anthocyanins to vacuoles (Kitamura et al., 2004), cation/H+ exchanger 3 influences color formation because of their effects on cellular pH and Na+/K+ homeostasis, and likely plays a crucial role in the blue color chang (Xu et al., 2022; Wang et al., 2025), potassium channel KAT3 directly affects the intensity of anthocyanin synthesis, hue transition and distribution uniformity by regulating cell turgor pressure, pH value and metabolic enzyme activity via K^+^ transport (Yan et al., 2025).

CYB561A, XTHB, ADT2, GPAT6, CTL1, and XTH1 were significantly enriched in “pigment accumulation in tissues” (**Supplementary File S14**). Except ADT2, other genes abundance in ZHs were more than that in BH, suggesting that increase of CYB561A, XTHB, GPAT6, CTL1, and XTH1 facilitate the accumulation of pigments. CYB561A affects the accumulation of anthocyanins, which are important for plant coloration, it helps regulate the amount and location of anthocyanin deposition, influencing the intensity and distribution of colors in flowers and fruits by maintaining the redox environment and participating in transporting certain pigments into vacuoles (Gradogna et al., 2023). TFs, such as MYB, AP2/ERF, bZIP, TCP, and GATA, were dramatically expressed and focused on the regulation of genes in the upstream synthesis of Phe (DAHPS; ADT/PDT) and the synthesis of AP (phenylacetaldehyde reductase; short-chain dehydrogenase/reductase), Dp (F3’H; F3’5’H), and DpG (BZ1), but inhibited the formation of flavones (flavonol synthase) and catechins (leucoanthocyanidin reductase) (Mei et al., 2021). Furthermore, GPAT6 is involved in cuticle synthesis to affect petal pigment deposition (Li-Beisson et al., 2009). Phenylalanine (Phe) is a precursor of flavonoids. ADTs the key enzymes that catalyze the conversion of arogenate into Phe in sucrose-induced anthocyanin biosynthesis in Arabidopsis. Among all six ADT isoforms, ADT2 contributes the most to anthocyanin accumulation, the level of Phe is an important regulatory factor for sustaining anthocyanin biosynthesis (Chen et al., 2016). However, this study indicate that the relative expression level of ADT2 was lower in purple flowers than in white flowers, whereas the relative expression levels of ADT1, ADT3 and ADT6 were higher in purple flowers than in white flowers, ADT4 and ADT5 were not identified among the total differentially expressed genes between BH and ZHs, suggestting that ADT2 may not play a major role in anthocyanin accumulation in alfalfa, whereas ADT1 might be the key contributors depending on relative expression abundance of ADT1, ADT3 and ADT6. Therefore, we speculate that different ADT isoforms may play dominant roles in anthocyanin accumulation across different plant species, or that these ADT isoforms possess distinct functional characteristics in different species.

### 4.6 Different purple color pattern formation

Different pigment types and their contents in spatio-temporal combination ultimately determine flower color (Tanaka et al., 2008; Grotewold, 2006). Anthocyanins are the crucial pigments that endow petals with various colors, and commonly include cyanidin, delphinidin, malvidin, pelargonidin, peonidin and petunidin (Santos Buelga et al., 2014).

The study revealed that the variations in the contents and proportions of pigment glycosides across white, light purple, purple, and deep purple flowers directly determine petal coloration. Specifically, deep purple flowers exhibited substantially higher contents and proportions of specific anthocyanin glycosides, whereas white flowers contain almost no anthocyanin glycosides. The proportional relationship between different pigment glycosides not only determines the hue of flower color but also affects its shade. For example, changes in the ratio of delphinidin glycosides to cyanidin glycosides shift flower color between blue and red tones. The differences in content and proportion are the final results of gene expression regulation and metabolic pathway regulation, which are closely related to the CSDEGs and metabolic pathways discussed earlier.

The main pigments that make alfalfa flower purple are the large amounts of blue delphinidin and red or purple malvidin, petunian, cyanidin, pelargonidin, paeonidin. From light purple to deep purple, the content of both blue pigment and red pigment showed a significant increase trend (**Table 2**), which is the reason for the gradual enrichment and saturation of flower color. According to the decreasing trend of the ratio of blue pigment and red pigment content in the flowers from light purple to deep purple, combined with the fact that the content of blue pigment is much higher than that of red pigment (**Table 2**), the overall color gradually shift from blue to red, or the blueness weaken as the ratio of the two pigments decreases. This indicates that flower color is dominated by the superimposition effect of delphinidin and malvidin derivatives. This result confirms the classic theory of “flower color is determined by pigment combination”, but the specific superimposition model needs to be further verified by combining optical properties.

A previous study suggested that malvidin and petunidin derivatives are the major anthocyanins found in the purple flowers of alfalfa, and in addition to malvidin and petunidin derivatives, cyanidin, pelargonidin, peonidin and delphinidin derivatives are also the essential anthocyanins in the petals of purple flowers (Duan et al., 2020). In contrast, Huang et al. (2024) identified delphinidin-3, 5-O-diglucoside was the predominant pigments accumulated in purple flowers. In the present study, the contents of delphinase-3, 5-O-digluboside and malvacin-3, 5-O-digluboside ranked first and second among 64 anthocyanins in purple flower and their content is absolutely dominant in the total anthocyanin content (**Tables 1**, **2**), and delphinase-3, 5-O-diglucoside is blue, malvin-3, 5-O-diglucoside is red, so it can be inferred that the purple color of alfalfa flower is mainly composed of and determined by the two pigments. Among them, delphinidin-3, 5-O-diglucoside plays a dominant role, which is consistent with the results of Huang et al., 2024, but contradict to the results of Duan et al., 2020. The main reason is that only delphinidin 3-O-glucoside, cyanidin 3-O-glucoside, pelargonidin 3-O-glucoside, peonidin 3-O-glucoside, malvidin 3-O-glucoside, and petunidin 3-O-glucoside were detected in Duan et al., 2020.

### 4.7 Constraints and suggestions for further investigation

Alfalfa is inherently a heterozygote, with distinct genetic backgrounds corresponding to different flower colors. Analysis of our transcriptomic and metabolomic data revealed that metabolomic profiles exhibited high consistency and reproducibility when flower color was strictly uniform, confirming that white flowers were suitable as the control group. In contrast, for transcriptomic data, although we had made every effort to ensure consistent flower color during sample collection, the consistency and reproducibility of transcriptomic profiles among samples of the same flower color were relatively low. Furthermore, even though we identified common differentially expressed genes by comparing gradient purple flower samples with white flower samples, the negative impacts caused by genetic background variations were not completely eliminated, resulting in transcriptomic outcomes that were less satisfactory than expected.

To address the issue of genetic background differences in heterozygous alfalfa, two solutions are proposed: (1) Expand sample types and population size while maintaining consistent phenotypic traits, and adopt mixed sampling strategies to reduce the interference of individual genetic variations. (2) Establish homozygous lines via consecutive generations of selfing purification or single-plant *in vitro* culture technology; additionally, create flower color mutants through EMS mutagenesis and use these homozygous lines and their corresponding mutants as research samples. This approach fundamentally resolves the heterozygosity problem and represents a long-term, thorough solution to improve the reliability of experimental data.

Recommendations for further investigation into the molecular mechanism of flower color formation are as follows: (1) Validate the functions and underlying mechanisms of the key uncharacterized genes identified in this study during flower color development using techniques including gene silencing/overexpression, yeast two-hybrid assay, and subcellular localization. (2) Improve and enrich the anthocyanin biosynthesis pathway by screening and identifying other enzymes directly involved in anthocyanin synthesis across multiple plant species using diverse strategies, methods, and technologies. (3) Compare and analyze alfalfa-specific regulatory genes by referencing the well-characterized flower color regulatory mechanisms of model plants (e.g., *Arabidopsis thaliana* and *Petunia hybrida*), thereby clarifying the species-specific characteristics of flower color formation in alfalfa.

## Data Availability

All raw sequence data have been submitted to the Genome Sequence Archive (GSA) database under accession number PRJCA055713. The addresses are as follows: https://ngdc.cncb.ac.cn/gsub/submit/gsa/subCRA064454/finishedOverview.

## Supplementary material

Supplementary File 1 The contents of 64 anthocyanins in BH, ZH1, ZH2, ZH3, and ZH4

Supplementary File 2 The contents of 68 carotenoids in BH, ZH1, ZH2, ZH3, and ZH4

Supplementary File 3 Genes assembled and mapped to the reference genome

Supplementary File 4 The SDEGs between BH and ZH1

Supplementary File 5 The SDEGs between BH and ZH2

Supplementary File 6 The SDEGs between BH and ZH3

Supplementary File 7 The SDEGs between BH and ZH4

Supplementary File 8 The TSDEGs between BH and ZHs

Supplementary File 9 CSDEGs between BH and the four ZH groups

Supplementary File 10 Sequences of five genes unannotated in NR database

Supplementary File 11 The expression trends of CSDEGs in BH, ZH1, ZH2, ZH3, and ZH4

Supplementary File 12 Transcription factors identified in the 174, 531 genes

Supplementary File 13 Pathway Enrichment Analysis result of CSDEGs

Supplementary File 14 GO Enrichment Analysis result of CSDEGs

Supplementary File 15 –34 important genes from CSDEGs not enriched in KEGG pathways

Supplementary File 16 Genes significantly strongly correlated with delphinidin-3, 5-O-diglucoside

Supplementary File 17 Genes significantly strongly correlated with malvidin-3, 5-O-diglucoside

Supplementary Table 1 The qPCR primer sequence
