# Supplementary material for: Integrated transcriptomic and metabolomic profiling reveals the flower color formation mechanism of alfalfa different purple flowers
Source: Front Plant Sci. 2026 Mar 23;17:1786493. doi: 10.3389/fpls.2026.1786493 (PMC13050952; doi:10.3389/fpls.2026.1786493)
Supplement: Supplementary File 15 — –34 important genes from CSDEGs not enriched in KEGG pathways [file Table15.docx]

| **No.** | **Gene category** | **Gene ID** | **Annotated Name** | **Function** | **Supporting Literature** |
| --- | --- | --- | --- | --- | --- |
| 1 | Key enzymes for pigment biosynthesis and modification | MS.gene03490 | cytochrome P450 82A3 | It may affect the pigment structure and flower color by participating in the hydroxylation or cyclization modification of flavonoids (e.g., anthocyanins). | Tanaka Y, Brugliera F. Flower colour and cytochromes P450. Philos Trans R Soc Lond B Biol Sci.2013;368(1612):20120432.doi:10.1098/rstb.2012.0432. |
| 2 |  | MS.gene033310 | cytochrome P450 71A1 |  |  |
| 3 |  | MS.gene059947 | cytochrome P450 71A1 |  |  |
| 4 |  | MS.gene06128 | cytochrome P450 monooxygenase CYP716A12 |  |  |
| 5 |  | MS.gene28131 | cytochrome p450 |  |  |
| 6 |  | MS.gene66255 | cytochrome P450 71D11 |  |  |
| 7 |  | MSTRG.50324 | cytochrome P450 family protein |  |  |
| 8 |  | MS.gene31540 | coumaroyl-CoA:anthocyanidin 3-O-glucoside-6''-O-coumaroyltransferase 1 | It is involved in the acylation of the 6'' position of the 3-O-glucose residue of anthocyanin. Also able to use flavonol 3-glucosides as the acyl acceptor.  It is a flower color stability enhancement factor that determines hue transition, enhances pigment stability and contributes to the development of bluish hues. | Yamazaki M, Yamagishi E, Gong Z, etc. Two flavonoid glucosyltransferases from Petunia hybrida: molecular cloning, biochemical properties and developmentally regulated expression. Plant Mol Biol. 2002; 48(4): 401 -11.doi:10.1023/a:1014043214943.  2.Suzuki H, Nakayama T, Yonekura-Sakakibara K, etc. cDNA cloning, heterologous expressions, and functional characterization of malonyl-coenzyme a:anthocyanidin 3-o-glucoside-6"-o-malonyltransferase from dahlia flowers. Plant Physiol. 2002;130(4):2142-51.doi:10.1104/pp.010447. |
| 9 |  | MSTRG.49897 | HXXXD-type acyl-transferase family protein | It may affect pigment modification by participating in the acyl transfer reaction. | Its functional prediction is based on the analysis of homologous enzyme families. |
| 10 |  | MSTRG.23854 | 4-coumarate--CoA ligase-like 6 | the different 4CL genes are likely to participate in different biosynthetic pathways leading to the various phenylpropanoid-derived metabolites that help create flavor and color | Kumar A , Ellis B E .4-Coumarate:CoA ligase gene family in Rubus idaeus : cDNA structures, evolution, and expression[J].Plant Molecular Biology,2003,51(3):327-340.DOI:10.1023/A:1022004923982. |
| 11 | Genes involved in transport and vacuolar compartmentalization | MS.gene020652 | heavy metal-associated isoprenylated plant protein 6 (HIPP6) | It may act as a glutathione S-transferase (GST) chaperone to participate in the transport of anthocyanins to vacuoles. | Kitamura S, Shikazono N, Tanaka A. TRANSPARENT TESTA 19 is involved in the accumulation of both anthocyanins and proanthocyanidins in Arabidopsis. Plant J.2004;37(1):104-14.doi:10.1046/j.1365-313x.2003.01943.x. |
| 12 |  | MS.gene008000 | cation/H+ exchanger 3 | influence color formation because of their effects on cellular pH and Na+/K+ homeostasis. likely plays a crucial role in the blue color chang | 1.Xu Q, Xia M, He G, etc. New insights into the influence of NHX-type Cation/H+ antiporter on flower color in Phalaenopsis orchids. J Plant Physiol.2022;279:153857.doi:10.1016/j.jplph.2022.153857.  2.Wang B, Wang H, Liu M,etc. The vacuole pH-related gene RcNHX2 affects flower color shift and Na+ homeostasis in roses. Plant Sci.2025;357:112476.doi:10.1016/j.plantsci.2025.112476. |
| 13 |  | MS.gene016286 | potassium channel KAT3 | It directly affects the intensity of anthocyanin synthesis, hue transition and distribution uniformity by regulating cell turgor pressure, pH value and metabolic enzyme activity via K⁺ transport. | Yan T, Song Z, Yu B, etc. Analysis of rabbiteye blueberry metabolomes and transcriptomes reveals mechanisms underlying potassium-induced anthocyanin production. Sci Rep.2025;15(1):7573.doi:10.1038/s41598-025-90060-w. |
| 14 | Core transcription regulatory factors | MS.gene053010 | myb-related protein 305 | It may directly activate the expression of anthocyanin synthesis genes. | 1.Borevitz JO, Xia Y, Blount J, etc. Activation tagging identifies a conserved MYB regulator of phenylpropanoid biosynthesis. Plant Cell.2000;12(12):2383-2394.doi:10.1105/tpc.12.12.2383.  2.Gonzalez A, Zhao M, Leavitt JM, etc. Regulation of the anthocyanin biosynthetic pathway by the TTG1/bHLH/Myb transcriptional complex in Arabidopsis seedlings. Plant J.2008;53(5):814-27.doi:10.1111/j.1365-313X.2007.03373.x.  3.Zhang S, Wang H, Wang T, etc. MdMYB305-MdbHLH33-MdMYB10 regulates sugar and anthocyanin balance in red-fleshed apple fruits. Plant J.2023;113(5):1062-1079.doi:10.1111/tpj.16100.  4.Liu, G. . (2010). Functional study of transcription factor MYB305 in tobacco flower development.. (Doctoral dissertation, Iowa State University). |
| 15 |  | MS.gene54772 | Myb DNA-binding domain protein | It contains the MYB domain and may be involved in flower color regulation. | Its functional prediction is based on the analysis of homologous enzyme families. |
| 16 |  | MSTRG.86353 | MYB-like transcription factor EOBII | It regulates the biosynthesis of flower fragrance volatiles, but often shares a co-regulatory network with the flower color pathway. | 1.Spitzer-Rimon B, Marhevka E, Barkai O, etc. EOBII, a gene encoding a flower-specific regulator of phenylpropanoid volatiles' biosynthesis in petunia. Plant Cell.2010;22(6):1961-76. doi:10.1105/tpc.109.067280.  2.Cna'ani A, Spitzer-Rimon B, Ravid J,etc. Two showy traits, scent emission and pigmentation, are finely coregulated by the MYB transcription factor PH4 in petunia flowers. New Phytol. 2015 Nov;208(3):708-14. doi: 10.1111/nph.13534. |
| 17 |  | MS.gene025894 | transcription factor bHLH13 | It can form the MBW complex together with MYB and WD40, activating downstream genes to participate in the regulation of pigment synthesis. | 1.Xi H, He Y, Chen H. Functional Characterization of SmbHLH13 in Anthocyanin Biosynthesis and Flowering in Eggplant. Horticultural Plant Journal,2021,7(1): 73-80.https://doi.org/10.1016/j.hpj.2020.08.006  2.Li S. Transcriptional control of flavonoid biosynthesis: fine-tuning of the MYB-bHLH-WD40 (MBW) complex. Plant Signal Behav.2014;9(1):e27522.doi:10.4161/psb.27522.  3.Li Y, Shan X, Gao R, etc. MYB repressors and MBW activation complex collaborate to fine-tune flower coloration in Freesia hybrida. Commun Biol. 2020;3(1):396.doi:10.1038/s42003-020-01134-6. |
| 18 |  | MS.gene033132 | BHLH transcription factor | It can form the MBW complex together with MYB and WD40, activating downstream genes to participate in the regulation of pigment synthesis. | 1.Li S. Transcriptional control of flavonoid biosynthesis: fine-tuning of the MYB-bHLH-WD40 (MBW) complex. Plant Signal Behav.2014;9(1):e27522.doi:10.4161/psb.27522.  2.Li Y, Shan X, Gao R, etc. MYB repressors and MBW activation complex collaborate to fine-tune flower coloration in Freesia hybrida. Commun Biol.2020;3(1):396.doi:10.1038/s42003-020-01134-6. |
| 19 |  | MS.gene059664 | transcription factor bHLH18 | It can form the MBW complex together with MYB and WD40, activating downstream genes to participate in the regulation of pigment synthesis. | 1.Li S. Transcriptional control of flavonoid biosynthesis: fine-tuning of the MYB-bHLH-WD40 (MBW) complex.Plant Signal Behav.2014;9(1):e27522.doi:10.4161/psb.27522.  2.Li Y, Shan X, Gao R, etc. MYB repressors and MBW activation complex collaborate to fine-tune flower coloration in Freesia hybrida. Commun Biol.2020;3(1):396.doi:10.1038/s42003-020-01134-6. |
| 20 |  | MS.gene063386 | NAC domain-containing protein 21/22 | It may regulate the accumulation of anthocyanins. | Morishita T, Kojima Y, Maruta T, etc. Arabidopsis NAC transcription factor, ANAC078, regulates flavonoid biosynthesis under high-light. Plant Cell Physiol.2009;50(12):2210-22.doi:10.1093/pcp/pcp159. |
| 21 |  | MS.gene49570 | BZIP transcription factor | It may play a positive role in anthocyanin accumulation. | Zhang Y, Han P, Zhao R, etc. Transcriptome and Metabolome Analyses Reveal the Mechanism of Color Differences in Pomegranate (Punica granatum L.) Red and White Petals.Plants (Basel). 2025;14(5):652. doi:10.3390/plants14050652. |
| 22 | Genes involved in signal and modification | MS.gene050199 | ethylene-responsive transcription factor ERF106 | It may compete for binding to bHLH factors to inhibit the formation of the MBW complex, reduce anthocyanin accumulation, lighten flower color (toward yellow/orange hues), and affect the pigmentation of fruits and floral organs. By influencing the distribution of phenylpropanoid metabolic flux, it alters the ratio of anthocyanins to volatiles, thereby indirectly affecting the intensity of flower color. | 1.Min T, Yin XR, Shi YN, etc. Ethylene-responsive transcription factors interact with promoters of ADH and PDC involved in persimmon (Diospyros kaki) fruit de-astringency.J Exp Bot.2012;63(18):6393-405.doi:10.1093/jxb/ers296. 2.Zhang J, Xu H, Wang N, etc. The ethylene response factor MdERF1B regulates anthocyanin and proanthocyanidin biosynthesis in apple. Plant Mol Biol.2018;98(3):205-218.doi:10.1007/s11103-018-0770-5.  3.Yin X, Yang H, Ding K, etc. PfERF106, a novel key transcription factor regulating the biosynthesis of floral terpenoids in Primula forbesii Franch. BMC Plant Biol.2024;24(1):851.doi:10.1186/s12870-024-05567-7.  4.Wang Z, Song G, Zhang F, etc. Functional Characterization of AP2/ERF Transcription Factors during Flower Development and Anthocyanin Biosynthesis Related Candidate Genes in Lycoris. Int J Mol Sci. 2023;24(19):14464.doi:10.3390/ijms241914464. |
| 23 |  | MS.gene037131 | ethylene-responsive transcription factor ERF017 | It may respond to ethylene signals to regulate flower color. | Wang Z, Song G, Zhang F, etc. Functional Characterization of AP2/ERF Transcription Factors during Flower Development and Anthocyanin Biosynthesis Related Candidate Genes in Lycoris. Int J Mol Sci. 2023;24(19):14464. doi:10.3390/ijms241914464. |
| 24 |  | MS.gene50585 | WRKY transcription factor 21 | It affects flower color by binding to W-box and regulating the metabolism of anthocyanins and carotenoids. | Guo C, Chen Y, Wu L,etc. Genome-Wide Identification of WRKY Transcription Factors in Lagerstroemia indica and Their Involvement in Color Formation. Physiol Plant.2025;177(3):e70328.doi: 10.1111/ppl.70328. |
| 25 |  | MS.gene26650 | auxin-induced protein 5NG4-like | Auxin often antagonizes anthocyanin synthesis; thus, it may negatively regulate flower color. | Wang YC, Wang N, Xu HF, etc. Auxin regulates anthocyanin biosynthesis through the Aux/IAA-ARF signaling pathway in apple. Hortic Res. 2018;5:59.doi: 10.1038/s41438-018-0068-4. |
| 26 |  | MS.gene013994 | Cyclin-like F-box | F-box proteins act as translational and post-translational regulators. F-box genes play an important role for translational and post-translational  network in anthocyanin biosynthesis. F-box gene downregulation increases the content of certain flavonoids, resulting in deep purple flower color. | 1.Jo YD, Ryu J, Kim YS,etc. Dramatic Increase in Content of Diverse Flavonoids Accompanied with Down-Regulation of F-Box Genes in a Chrysanthemum (Chrysanthemum × morifolium (Ramat.) Hemsl.) Mutant Cultivar Producing Dark-Purple Ray Florets. Genes (Basel).2020;11(8):865.doi: 10.3390/genes11080865.  2.Liu X, Du F, Sun L,etc. Anthocyanin metabolism in Nelumbo: translational and post-translational regulation control transcription. BMC Plant Biol. 2023;23(1):61. doi: 10.1186/s12870-023-04068-3. |
| 27 |  | MSTRG.93661 | cyclin-like F-box protein |  |  |
| 28 |  | MS.gene003871 | F-box protein SKIP16 |  |  |
| 29 |  | MS.gene013995 | F-box protein At2g26850 |  |  |
| 30 |  | MS.gene03752 | F-box/LRR-repeat protein At4g14103 |  |  |
| 31 |  | MS.gene071943 | F-box/kelch-repeat protein At1g22040 |  |  |
| 32 |  | MS.gene43475 | F-box/kelch-repeat protein At3g06240 |  |  |
| 33 |  | MS.gene056338 | lysine-specific demethylase JMJ30 isoform X1 | It may participate in photoperiod regulation and crosstalks with the light-induced anthocyanin synthesis pathway. | Lu SX, Knowles SM, Webb CJ, etc. The Jumonji C domain-containing protein JMJ30 regulates period length in the Arabidopsis circadian clock. Plant Physiol.2011;155(2):906-15.doi:10.1104/pp.110.167015. |
| 34 | Genes involved in RNA regulation and protein modification and degradation | MS.gene007765 | protein argonaute 5 | It affects the expression level of flavonoid synthases by regulating the mRNA stability and translation efficiency of flower color-related genes. | Mach J. Saddle Up, Soybean Seed Pigments: Argonaute5 in Spatially Regulated Silencing of Chalcone Synthase Genes. Plant Cell. 2017;29(4):604. doi: 10.1105/tpc.17.00291. |
