# Supplementary material for: Integrated transcriptomic and metabolomic profiling reveals the flower color formation mechanism of alfalfa different purple flowers
Source: Front Plant Sci. 2026 Mar 23;17:1786493. doi: 10.3389/fpls.2026.1786493 (PMC13050952; doi:10.3389/fpls.2026.1786493)
Supplement: Supplementary Table 1 — The qPCR primer sequence [file Supplementaryfile3.docx]

| Primer table | |
| --- | --- |
| primer name | primer sequence |
| MS.gene049847F | CGTCCAATCTCCACCGTCAATCTC |
| MS.gene049847R | TTAGAAGCGTGAATGCGGAGGTTAC |
| MS.gene02084F | TCTTCCGGCTCCATTCAATTCCTTG |
| MS.gene02084R | TTCCAAATGTATGAGCACCCGAGAG |
| MS.gene033730F | ACCAACAGTGAGCACATGAC |
| MS.gene033730R | AGTGATGGTGCCATGTATGC |
| MS.gene069129F | TACTGATTTGGCTGCTGAGTTTGGG |
| MS.gene069129R | TGCGATCCATGTCCTCGTCTCC |
| S.gene71667F | GCGAGCTGCTAACAACATCATTGAC |
| MS.gene71667R | TGAAGGCCAAACCATCACCATCTG |
| MS.gene89382F | TGCTTCCACCATCCCTGCAAATAAG |
| MS.gene89382R | GACTCGCTGCCCAATCCTATGAAC |
| MS.gene91596F | TGTGCCTATTCGCTTGTTTGTTTGG |
| MS.gene91596R | AATGGTGGTTCTAACCTTGCTCTGC |
| MS.gene91614F | ACCCTCCCAAATTCACAATCACACC |
| MS.gene91614R | TCAGAAGGCCAAACGATGTTACCAG |
| MS.gene029004F | CATTCCTGCTGATCGTGTACCTGAC |
| MS.gene029004R | GAAGAACCTGAGCCTGGTGGAAC |
| MS.gene027054F | CGCCGAAACCCTCCATTCCATC |
| MS.gene027054R | GTTGTTGAATCCGCCGCATAAGC |
| MS.gene017490F | AAGGAGGGAAGAACATGGCTAC |
| MS.gene017490R | AGCTGCCTTGACAACAAAGG |
| MS.gene071930F | AAACCTCGTCGACCCAGAATG |
| MS.gene071930R | TGCCCACCTTCCATGAATAAGC |
| MSTRG.76939F | CGGTGAATTGGAGTTTGGCG |
| MSTRG.76939R | CAGCAGGAAGAGCGTGAGAA |
| GAPDH-F | TGGGAAGCACATTACAGCAG |
| GAPDH-R | CATCAGCATTGACACCAACC |
